# Light-independent pathway of STN7 kinase activation under low temperature stress in runner bean (*Phaseolus coccineus* L.)

**DOI:** 10.1186/s12870-024-05169-3

**Published:** 2024-06-07

**Authors:** Małgorzata Krysiak, Anna Węgrzyn, Łucja Kowalewska, Anna Kulik, Monika Ostaszewska-Bugajska, Jan Mazur, Maciej Garstka, Radosław Mazur

**Affiliations:** 1 Department of Metabolic Regulation, Institute of Biochemistry, Faculty of Biology, University of Warsaw, Miecznikowa 1, Warsaw, 02-096 Poland; 2 Department of Plant Anatomy and Cytology, Institute of Plant Experimental Biology and Biotechnology, Faculty of Biology, University of Warsaw, Miecznikowa 1, Warsaw, 02-096 Poland; 3 Institute of Biochemistry and Biophysics, Polish Academy of Sciences, Pawińskiego 5a, Warsaw, 02-106 Poland; 4 Department of Plant Bioenergetics, Institute of Plant Experimental Biology and Biotechnology, Faculty of Biology, University of Warsaw, Miecznikowa 1, Warsaw, 02-096 Poland; 5 Present Address: Institute of Biological Chemistry, Washington State University, Pullman, WA 99164 USA

**Keywords:** Abiotic stress, Chloroplast, Dark-chilling, LHCII phosphorylation, Runner bean, STN7 kinase, Thylakoids

## Abstract

**Background:**

The phosphorylation of the Light-Harvesting Complex of photosystem II (LHCII) driven by STATE TRANSITION 7 (STN7) kinase is a part of one of the crucial regulatory mechanisms of photosynthetic light reactions operating in fluctuating environmental conditions, light in particular. There are evidenced that STN7 can also be activated without light as well as in dark-chilling conditions. However, the biochemical mechanism standing behind this complex metabolic pathway has not been deciphered yet.

**Results:**

In this work, we showed that dark-chilling induces light-independent LHCII phosphorylation in runner bean (*Phaseolus coccineus* L.). In dark-chilling conditions, we registered an increased reduction of the PQ pool which led to activation of STN7 kinase, subsequent LHCII phosphorylation, and possible LHCII relocation inside the thylakoid membrane. We also presented the formation of a complex composed of phosphorylated LHCII and photosystem I typically formed upon light-induced phosphorylation. Moreover, we indicated that the observed steps were preceded by the activation of the oxidative pentose phosphate pathway (OPPP) enzymes and starch accumulation.

**Conclusions:**

Our results suggest a direct connection between photosynthetic complexes reorganization and dark-chilling-induced activation of the thioredoxin system. The proposed possible pathway starts from the activation of OPPP enzymes and further NADPH-dependent thioredoxin reductase C (NTRC) activation. In the next steps, NTRC simultaneously activates ADP-glucose pyrophosphorylase and thylakoid membrane-located NAD(P)H dehydrogenase-like complex. These results in starch synthesis and electron transfer to the plastoquinone (PQ) pool, respectively. Reduced PQ pool activates STN7 kinase which phosphorylates LHCII. In this work, we present a new perspective on the mechanisms involving photosynthetic complexes while efficiently operating in the darkness. Although we describe the studied pathway in detail, taking into account also the time course of the following steps, the biological significance of this phenomenon remains puzzling.

**Supplementary Information:**

The online version contains supplementary material available at 10.1186/s12870-024-05169-3.

## Background

The thylakoid network, a three-dimensional system of internal chloroplast membranes, is a space where photosynthetic light reactions take place. The main components of photosynthetic apparatus are multisubunit chlorophyll-protein (CP) complexes: photosystem II (PSII), the cytochrome b_6_f complex (Cyt b_6_f), and photosystem I (PSI). They are connected electrochemically by the mobile electron carriers: plastoquinone (PQ) and plastocyanin. Most of the photons used in the light reactions of photosynthesis are harvested by the Light-Harvesting Complex II (LHCII), which can act as an outer antenna of both photosystems. Switching of the LHCII pool between PSII and PSI serves as an adjustment mechanism that regulates the amount of excitation energy distributed to the reaction centers [1]. This mechanism, called state transitions, is regulated via reversible phosphorylation of LHCII, which is catalyzed by the STATE TRANSITION 7 (STN7) kinase [2]. The illumination that favors the excitation of PSII, leads to a high reduction of the PQ pool and activation of STN7 kinase [3]. STN7 phosphorylates Lhcb1 and Lhcb2 subunits of LHCII [1], which results in the dissociation of phosphorylated LHCII (P-LHCII) from PSII and energy distribution towards PSI (state 2). Contrarily, upon illumination that favors the excitation of PSI resulting in PQ oxidation, the P-LHCII pool gets dephosphorylated by the constitutively active thylakoid phosphatase TAP38/PPH1, which leads to reassociation of LHCII to PSII (state 1) [4, 5]. In a classical view of state transitions, in state 2, P-LHCII migrates through the thylakoid membrane towards unstacked membrane margins where PSI is located [6–8], to associate with PSI forming the PSI-LHCI-LHCII complex [9, 10]. However, recent discoveries suggest that the PSI-LHCI-LHCII complex is formed using the LHCII pool already present in unstacked membranes, thus migration of P-LHCII through thylakoid membrane is limited [11, 12].

From known posttranslational modifications of LHCII such as phosphorylation, acetylation [13], and polyamination [14] the first one plays the dominating role in the induction of CP complexes rearrangements under fluctuating environmental conditions. Light quality and intensity are the main factors influencing the activity of STN7 kinase. However, several reports show its activation under dark conditions. In our previous work, we reported an increased LHCII phosphorylation in *Arabidopsis thaliana* under dark conditions [15]. Non-photochemical reduction of PQ, which activates STN7 kinase in the dark, was reported for Arabidopsis to be mediated by Proton Gradient Regulator 5 (PGR5) independent antimycin A sensitive pathway [16] and by NAD(P)H dehydrogenase-like complex (NDH) in dark-anaerobic conditions [17]. It was also reported that temperature influences STN7 kinase activation in the dark. LHCII phosphorylation was observed under moderate heat stress (40 °C) in the dark in wheat [18] and Arabidopsis [19]. Moreover, low temperature in the dark caused an increase in the LHCII phosphorylation in the runner bean [20]. LHCII phosphorylation under dark-chilling conditions seems to be a species specific phenomenon, as for garden pea a contradictory effect was observed (decrease in LHCII phosphorylation under dark-chilling) [20]. Interestingly, in Arabidopsis only a transient increase was detected [15, 19, 21].

Plant response to low-temperature is a complex process that influences plant metabolism on all levels of organization. On the chloroplast level, low temperature causes the reduction of photochemical reactions capacity, reorganization/degradation of CP complexes [22, 23], the decrease of Calvin-Benson-Bassham cycle enzymes activity, lipid peroxidation, and the increase of ROS level [reviewed in: 24, 25–27]. Consequently, these lead to the imbalance of chloroplast redox poise. In chloroplast, thioredoxin systems play a central role in the detection and signaling of the redox status [reviewed in 28]. The ferredoxin-thioredoxin system, relies on reducing equivalents generated in the chloroplast electron transport chain to supply electrons from reduced ferredoxin to various thioredoxins through ferredoxin-thioredoxin reductase [reviewed in 29, 30]. Other system involves NADPH-dependent thioredoxin reductase C (NTRC) [31]. NTRC is reduced by NADPH generated both in the chloroplast electron transport chain in the light and oxidative pentose phosphate pathway (OPPP) in the dark and under low irradiance [31, 32]. There are evidences that NTRC activity is related with LHCII phosphorylation as Arabidopsis mutant overexpressing NTRC presents LHCII phosphorylation even in the dark [33]. Also, NTRC has been suggested to regulate cold response. The work of Moon et al. [34] showed that NTRC overexpression enhances Arabidopsis tolerance to freezing and low temperatures. Contradictorily, it was proved that the transcript level of NTRC decreases during 24 h of chilling stress in Arabidopsis [35]. However, Chae et al. [36] reported that both cold and heat induce the *NTRC* transcript, while dark and extended night had an opposite effect.

In this study we characterized the light-independent STN7 kinase activation pathway which operates in runner bean under dark-chilling conditions. These results provide a new perspective on the mechanisms involving photosynthetic complexes while efficiently operating in the darkness.

## Materials and methods

### Plant growing conditions and dark-chilling treatment

Runner bean (*Phaseolus coccineus* L. cv. Eureka) plants (seeds from PlantiCo Zielonki, 05–082 Babice Stare, Poland) were grown in perlite-containing pots in a climate-controlled room in 16 h day/8 h night photoperiod (21 °C day/20°C night) at PAR of 200 µmol photons m^− 2^ s^− 1^ until fully grown. Fully expanded primary leaves of 10 days-old plants were collected at the end of the night period before the light was turned on. Dark-chilling treatment was applied as described before [37]. In short, detached leaves were placed in Dewar flasks (4 °C, 100% relative humidity) for three days. For control conditions plants were kept in darkness at 21 °C for three days (dark) or in a growth chamber in day/night cycle conditions (light).

### Preparation of leaf protein extracts, thylakoid membranes, and thylakoid fractions

Around 150 mg of leaf samples were frozen in liquid nitrogen, ground in a mortar to a powder, and transferred to the extraction buffer (50 mM Tris-HCl (pH 8.0), 10% (v/v) glycerol, 2% (w/v) SDS, 25 mM EDTA, 1 mM phenylmethylsulfonylfluoride), mixed and immediately frozen in liquid nitrogen. Frozen samples were placed in an ultrasonic bath at 4 °C until complete melting, then frozen again. Three freezing-thawing cycles were performed, then samples were centrifuged at 10,000 g to remove non-soluble material. Protein concentration in the supernatant was estimated according to the modified Lowry method using BioRad DC Protein Assay (#5000111).

Thylakoid membranes were isolated as described previously [38]. Leaves were ground in a Warring homogenizer in 20 mM Tricine-NaOH buffer (pH 7.5) containing 330 mM sorbitol, 40 mM ascorbic acid, 15 mM NaCl, 4 mM MgCl_2_, and 10 mM NaF. The homogenate was filtered through Miracloth and the filtrate was centrifuged for 4 min at 2000 g. The supernatant was discarded, and the pellet of chloroplasts was subjected to osmotic shock by suspending in 20 mM Tricine-NaOH buffer (pH 7.5) containing 15 mM NaCl, 4 mM MgCl_2_, and 10 mM NaF followed by centrifugation for 10 min at 6000 g. The resulting pellet of thylakoid membranes was suspended in 20 mM Hepes-NaOH buffer (pH 7.0) containing 330 mM sorbitol, 15 mM NaCl, 4 mM MgCl_2_, and 10 mM NaF and centrifuged again for 10 min at 6000 g. The pellet containing thylakoid membranes was suspended in a Potter-Elvenhjem homogenizer in a small volume of the same buffer. The concentration of chlorophyll (Chl) was quantified on a spectrophotometer using 80% (v/v) acetone extracts [39].

To separate thylakoid fractions the modified protocols described by Barber et al. [40] and Fristedt et al. [41] were used. Thylakoid samples were diluted in 20 mM Hepes-NaOH buffer (pH 7.0) containing 330 mM sorbitol, 15 mM NaCl, 4 mM MgCl_2_, and 10 mM NaF to 60 µg Chl mL^− 1^ concentration and 4% (w/v) digitonin was added to 0.11% (w/v) final concentration [40]. Samples were incubated in dark for 30 min at 4 °C with mild shaking [40], then centrifuged at 1000 *g* at 4 °C for 5 min in order to remove non-solubilized thylakoids [41]. The supernatant was diluted 6 times in the buffer described above and centrifuged at 40 000 *g* at 4 °C for 30 min [41]. The pellet containing stacked membranes corresponding to the so-called grana fraction (grana core and grana margins) was re-suspended in a small amount of dilution buffer, while the supernatant was centrifuged again at 100 000 *g* at 4 °C for 2 h. The pellet containing stroma lamellae fraction was re-suspended in a small amount of dilution buffer, and Chl content in both grana and stroma lamellae samples was assessed using 80% (v/v) acetone extracts.

### SDS PAGE and immunoblotting

Samples containing 10 µg of protein/1 µg of Chl were denatured in Laemmli buffer for 5 min at 75 °C, loaded onto polyacrylamide gels and separated by the standard SDS PAGE protocol using Tris-glycine running buffer. Proteins were transferred for 45 min at 100 V onto a polyvinylidene fluoride (PVDF) membrane in a buffer consisting of 192 mM glycine, 10% (v/v) methanol, and 25 mM Tris (pH 8.3). Membranes were blocked in 20 mM Tris-HCl (pH 7.5), 0.5 M NaCl buffer (TBS) with 3% (w/v) bovine serum albumin (Sigma-Aldrich) for 1 h at room temperature, washed with TBS and incubated overnight at 4 °C with primary antibodies (Agrisera, Vännäs, Sweden; list of used primary antibodies with catalog numbers is given in Table S1) diluted in TBST (TBS supplemented with 0.1% (v/v) Tween 20), accordingly to the manufacturer’s instructions. The next day, membranes were washed 4 times in TBST and incubated with anti-rabbit HRP-conjugate secondary antibody (Agrisera Catalog Number AS09 602) for 1 h at room temperature. Visualization was obtained by using ECL Bright (Agrisera Catalog Number AS16 ECL-N-100).

### Two layer Phos-Tag™ SDS PAGE

Two-layer Phos-Tag™ SDS PAGE was performed according to the method of Longoni et al [42]. Two-layer separating gels solutions were prepared: (i) heavy gel solution: 357 mM Bis-Tris (pH 6.8), 7% (w/v) acrylamide/bis-acrylamide (37.5:1), 30% (w/v) glycerol, 0.05% (v/v) N, N,N’,N’-tetramethylethylenediamine (TEMED); (ii) light gel solution: 357 mM Bis-Tris (pH 6.8), 8% (w/v) acrylamide/bis-acrylamide (37.5:1), 60 µM Phos-tag™ (FUJIFILM Wako Pure Chemical Corporation, Japan), 0.01% (w/v) Coomassie Brilliant Blue G-250, 0.05% (v/v) TEMED, 0.05% (w/v) APS. Three volumes of heavy solution were poured between the gel plates, followed by one volume of light solution. After polymerization, the stacking gel was prepared: 357 mM Bis-Tris (pH 6.8), 4% (w/v) acrylamide/bis-acrylamide (37.5:1), 0.1% (v/v) TEMED, 0.05% (w/v) APS.

Thylakoid samples were incubated for 5 min at 70 °C in a loading buffer containing 244 mM Tris HCl (pH 8.5), 2% (w/v) LDS, 10% (w/v) glycerol, 100 mM DTT, 0.33 mM Coomassie Brilliant Blue G-250. One µg of Chl was loaded per well. Electrophoresis was carried out with freshly prepared running buffer (61 mM Tris, 50 mM MOPS, 0.1% (w/v) SDS, 5 mM sodium bisulfite) at 55 V for 4.5 h. Then proteins were transferred onto a PVDF membrane in a buffer consisting of 25 mM Bis-Tris, 25 mM Bicine, 1 mM EDTA, 10% (v/v) methanol, 5 mM sodium bisulfite; pH 7.2, for 16.5 h at 150 mA at 4 °C. The membranes were blocked in TBS buffer containing 3% (w/v) bovine serum albumin, 0.1% (v/v) Triton X-100 for 40 min with agitation, then dephosphorylated by incubation with 400 U mL^− 1^ of λ protein phosphatase (New England BioLabs, catalog number P0753L), 2 mM MnCl_2_, 2 mM DTT in a blocking solution for 4 h in room temperature. The membranes were then washed with TBS and incubated with primary antibodies against Lhcb1 and Lhcb2 (Agrisera) diluted in TBST accordingly to the manufacturer’s instructions. Further steps of immunodetection were performed as described above. Phosphorylation level was calculated as [P/(P + NP)] × 100% (P, phosphoprotein; NP, unphosphorylated protein) and normalized to 0% at time point 0 h (i.e. the % of phosphorylation at 0 h was subtracted from data for all time points).

### Blue native PAGE and 2D electrophoresis

Blue native PAGE (BN-PAGE) was performed according to Rogowski et al. [43] with some modifications. Thylakoid samples (45 µg Chl) were centrifuged at 7000 *g* for 5 min at 4 °C, and the pellet was resuspended in a buffer containing 25 mM Imidazole-HCl (pH 7.0) and 20% glycerol. Thylakoid membrane proteins were solubilized by the addition of n-dodecyl-β-D-maltoside (DDM) to a final concentration of 1% (w/v). Samples were shaken vigorously and centrifuged at 18 000 *g* for 15 min at 4 °C. Sample buffer (50 mM Imidazole-HCl (pH 7.0), 125 mM 6-aminohexanoic acid, 30% (w/v) sucrose, 5% (w/v) Coomassie Brilliant Blue G-250) was added to the supernatant to a final concentration of 20%. The sample was loaded directly onto the gel (4–10% acrylamide, 4–15% sucrose gradient). Electrophoresis was performed overnight at constant 85 V at 4 °C using cathode buffer (50 mM Tricine, 7.5 mM Imidazole, 0.02% (w/v) Coomassie Brilliant Blue G-250) and anode buffer (25 mM Imidazole-HCl, pH 7.0). Alternatively, thylakoid membrane proteins were solubilized in 1% (w/v) digitonin during 5 min in dark at 20 °C with gentle mixing [44]; other steps were the same as for DDM.

Bands cut out from Blue Native PAGE gels were denatured in 125 mM Tris-HCl (pH 6.8), 5 M urea, 10% (v/v) glycerol, 5% (w/v) SDS, and 5% (v/v) β-mercaptoethanol for 15 min at room temperature followed by 15 min at 50 °C. Water-washed bands were loaded onto polyacrylamide gels, sealed with 1% (w/v) agarose, and separated by the standard SDS PAGE protocol using Tris-glycine running buffer. Gels were silver-stained according to [45].

### Protein digestion and LC-MS/MS

Silver-stained bands were excised, de-stained with 15 mM potassium hexacyanoferrate (III), 50 mM sodium thiosulphate solution, and subjected to in-gel protease digestion using trypsin in 100 mM ammonium bicarbonate overnight at 30 °C. After extraction from gel pieces, peptides were dried in a SpeedVac concentrator, dissolved in 1% (v/v) trifluoroacetic acid, 5% (v/v) acetonitrile mixture, and separated using a nanoAcquity Ultra Performance LC system connected to a Synapt G2 HDMS mass spectrometer (Waters). Peptides were trapped on Symmetry® C18 (5 μm; 180 μm × 20 mm) column (Waters) and separated on a BEH 130 C18 analytical column (1.7 μm; 75 μm × 200 mm) (Waters). The elution was performed with a linear gradient of acetonitrile in 0.1% formic acid. Protein identification was performed using ProteinLynx Global Server (PLGS version 2.4) software (Waters, Milford, MA, USA).

### Low-temperature chlorophyll *a *fluorescence (77 K) measurements

Steady-state fluorescence emission spectra of Chl at low temperature (77 K) were collected using a modified Shimadzu RF-5301PC spectrofluorometer as described previously [20]. Thylakoid membranes were diluted to 10 µg Chl mL^− 1^ concentration, placed in a polytetrafluoroethylene cuvette, and submerged in liquid nitrogen. The excitation wavelength was set at 440 nm, excitation, and emission slits at 5 nm, and scans were taken in the range of 600 to 800 nm (1 nm interval) through the LP600 emission filter.

### In vivo Chlorophyll *a *fluorescence measurements

The slow kinetics of Q_A_ reduction was measured in RT using a Dual PAM 100 fluorometer (Heinz Walz GmbH, Effeltrich, Germany) in the presence of blue measuring light of intensity below 0.5 µmol photons m^–2^ s^–1^ and red actinic light of 3 µmol photons m^–2^ s^–1^ intensity during one minute. PSII fluorescence increases, when there is a lack of downstream electron acceptors; so when the PQ pool is highly reduced, it cannot accept electrons from Q_A_, which results in an increased PSII fluorescence.

Non-photochemical PQ reduction activity was measured according to [46] with some modifications using the Maxi version of the PAM-Imaging system. After F_0_ and F_M_ determination and 60 s dark recovery the blue actinic light of 390 µmol photons m^–2^ s^–1^ intensity was on for 5 min. Then the actinic light was turned off and the signal was monitored for 5 min more. An increase of Chl *a* fluorescence level in the dark was an indicator of non-photochemical PQ reduction.

### Photosynthetic complex quantification

Photosynthetic complex quantification was carried out as described by [47]. Thylakoid samples (50 µg Chl mL^− 1^) were destacked by incubation for at least 10 min with 0.03% (w/v) DDM. Then, 1 mM potassium hexacyanoferrate (III) was added to fully oxidize all cytochromes. After that, the high-potential form of cyt b_559_ and cytochrome f were reduced by incubation with 10 mM sodium ascorbate. Then, 10 mM sodium dithionite was added to reduce the low potential form of cyt b_559_ and the two b-type hemes of cytochrome b_6_. At each of the three redox potentials, absorbance spectra were measured using a V-550 spectrophotometer equipped with a head-on photomultiplier (Jasco GmbH). Spectra were measured between 540 and 575 nm, 1 nm spectral bandwidth, and 100 nm min^–1^ scanning speed. Average from ten spectra per redox condition was used to calculate difference spectra: the hexacyanoferrate spectrum was subtracted from the ascorbate spectrum, and the ascorbate spectrum was subtracted from the dithionite spectrum, respectively, and baseline was subtracted from spectra. Finally, the difference spectra were deconvoluted using reference spectra, and the contents of PSII and the cyt b_6_f were determined as described by [48].

PSI content was calculated from light-induced difference absorbance changes of P700. Thylakoid samples (50 µg Chl mL^− 1^) were destacked by incubation with 0.2% (w/v) DDM. Then, the electron donor (10 mM sodium ascorbate) and electron acceptor (100 µM methyl viologen) were added, and a light pulse of 250 ms (2000 µmol photons m^–2^ s^–1^) was applied. P700 photo-oxidation was measured using the Pc-P_700_ version of the Dual-PAM instrument (Heinz Walz GmbH).

### Transmission electron microscopy

For transmission electron microscopy samples of 3 mm^2^ were cut from the middle parts of the leaves and prepared as described previously [49]. The 70 nm thick sections were examined with a JEM 1400 electron microscope (Jeol, Japan) equipped with Morada G2 (EMSIS GmbH) CCD camera in the Laboratory of Electron Microscopy, Nencki Institute of Experimental Biology of PAS (Warsaw, Poland).

### Metabolites analysis

#### PQ and PQH_2_

Prenyl lipid fraction (containing PQ and PQH_2_) was extracted and analyzed as described by [50]. Samples containing 200–300 mg of leaf were ground in cooled to -20 °C ethyl acetate. Extracts were transferred to Eppendorf tubes, evaporated under an argon stream, and stored at -80 °C for a maximum of two weeks before analysis. Extracts were separated using the Shimadzu Prominence HPLC System with Beckman Coulter ODS analytical column (5 μm, 4.6 mm × 250 mm). Right before separation samples were reconstituted in 400 µL of methanol: hexane (340:20, v/v) mixture, centrifuged at 10,000 g for 30 s, and the 100 µL of supernatant was injected into Rheodyne™ 7725i sample injector. Isocratic separation was performed using methanol: hexane (340:20, v/v) at 30 °C and mobile phase flow of 1.5 mL min^− 1^ for 40 min. Elution was monitored with SPD-M20A PDA detector (1.2 nm spectral resolution) and RF-10 A XL fluorescence (Ex 290 nm, Em 330 nm) detector. PQ and PQH_2_ contents were calculated based on peak areas extracted from chromatograms (absorption at 255 nm for PQ, fluorescence for PQH_2_) and comparison with prepared standards. PQ and PQH_2_ standards concentration were calculated spectrophotometrically using absorption coefficients: PQ ε_255_ = 17.94 mM^− 1^cm^− 1^; PQH_2_ ε_290_ = 3.39 mM^− 1^cm^− 1^; % of PQ reduction was calculated using following formula: PQ_red_ = [PQH_2_ / (PQ + PQH_2_)] × 100%.

### Starch

Starch content was quantified using the protocol of [51]. Leaves were frozen in liquid nitrogen, transferred to 80% (v/v) ethanol, and incubated at 100 °C for 3 min. Leaf tissue was then sedimented at 9 000 *g* for 5 min. The supernatant was discarded and ethanol extraction was repeated twice more. Pellet was homogenized in distilled water, then heated at 100 °C for 10 min. 200 mM sodium acetate (pH 5.5), 6 units of α -amyloglucosidase (Sigma-Aldrich, Cat. No. 10115), and 1.5 units of α–amylase (Sigma-Aldrich, Cat. No. A6380) were added to cooled homogenate and samples were incubated at 37 °C overnight. Then samples were assayed for glucose using an enzymatic assay with hexokinase (Sigma-Aldrich, Cat. No. H4502) and glucose 6-phosphate dehydrogenase (Sigma-Aldrich, Cat. No. G6378). NADPH production was measured at 340 nm on a Cary50Bio spectrophotometer.

### NADPH/NADP^+^

Pyridine nucleotides were extracted from leaf tissue as described by [52]. Briefly, tissues were homogenized with 0.1 M HCl (oxidized form of nucleotides) or 0.1 M NaOH (reduced form of nucleotides) in 50% (v/v) ethanol. Alkaline extracts were heated at 65 °C for 15 min and then cooled on ice. The homogenates were centrifuged at 14 000 *g* for 20 min at 4 °C. Phosphorylated pyridine nucleotides concentration in extracts was measured in 60 mM Bicine (pH 7.8) by enzymatic cycling reaction according to [53 and references therein] with spectrophotometric detection and the results were quantitated by comparison with a standard curve.

### **Enzyme activity measurements**

OPPP enzyme activities were measured according to the method of [54]. Leaves were ground in liquid nitrogen, and samples were extracted in a buffer containing 50 mM Tris–HCl (pH 8.0), 100 mM NaCl, 0.1 mM phenylmethylsulfonylfluoride, and 0.1 mM benzamidine. Homogenates were centrifuged at 10 000 *g* for 10 min at 4 °C. Supernatants were immediately used to measure enzyme activity. The 6-phosphogluconate dehydrogenase (6PGDH) activity was measured in a reaction buffer consisting of 100 mM HEPES, 0.4 mM NADP^+^, and 3 mM 6-phosphogluconic acid. The glucose-6-phosphate dehydrogenase (G6PDH) activity was measured in a buffer consisting of 100 mM Tris–HCl (pH 8.0), 0.4 mM NADP^+^, and 2 mM glucose 6-phosphate. NADPH production was monitored at 340 nm using a Cary50Bio spectrophotometer.

### RT-qPCR analysis

Leaves were ground to a fine powder in liquid nitrogen. RNA was extracted with Trizol reagent (Molecular Research Center) according to the manufacturer’s instructions and treated with DNase 1 (Thermo Scientific). Reverse transcription was performed on 1 µg of RNA using the RevertAid First Strand cDNA Synthesis Kit (Thermo Scientific). The resulting cDNA was diluted 10-fold with water and 1 µL of the sample was assayed by qPCR in a Step One Plus device (Applied Biosystems) using GoTaq® qPCR Master Mix (Promega) and specific pairs of primers (Table S2). To design primers for NTRC fragment amplification from runner bean the AT2G41680 cds nucleotide sequence was blast against the *Phaseolus vulgaris* genome and the Phvul.007G251800 (XM_007145532.1) sequence was selected to design primers. Four primer pairs covering different exon junctions were tested by RT-qPCR giving the same results of NTRC gene expression in runner bean and one of them was selected for further detailed analyses (Table S2). The relative expression level of NTRC was calculated using the delta-delta Ct method and normalized to the housekeeping genes *PvSkip16* (Phvul.011G053400), and *PvIDE* (Phvul.001G133200) according to [55]. Four independent biological replicates were performed using leaves from 3 to 4 plants per sample.

### Statistical analysis

The statistical significance was verified by one-way ANOVA with post hoc Tukey test at *p* = 0.05. The number of repetitions of specific experiments is given in the figure legends.

## Results

### Dark-chilling induces phosphorylation of LHCII proteins in runner bean

**Fig. 1:**
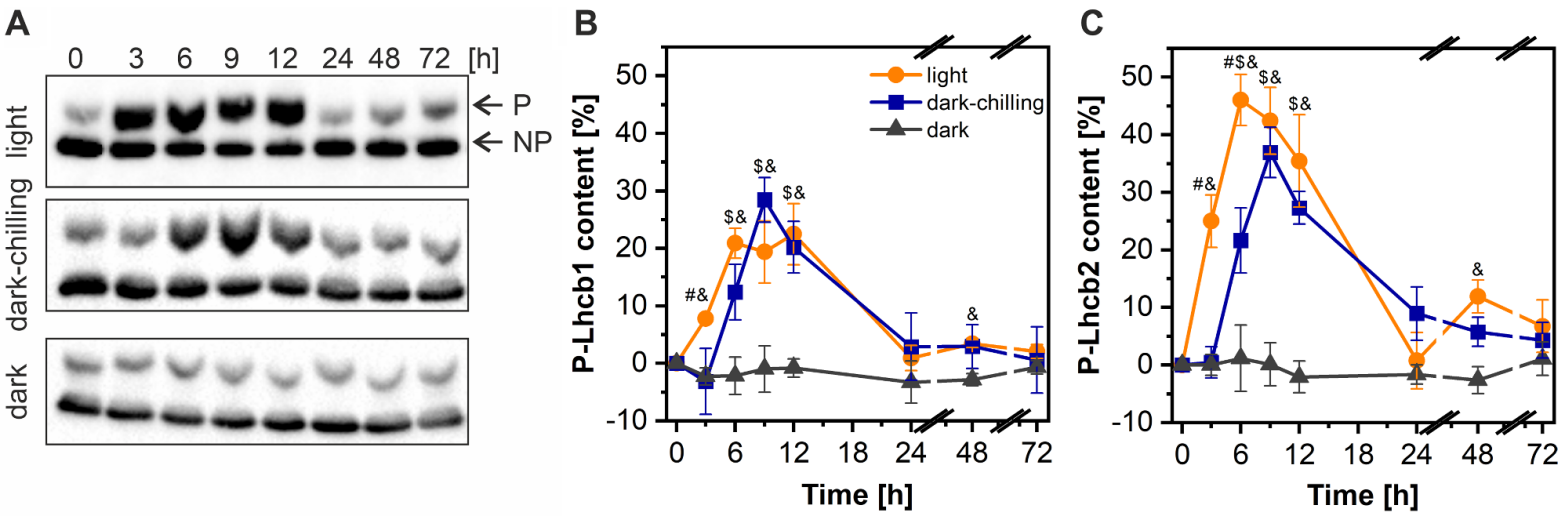
Dark-chilling increases LHCII phosphorylation in runner bean plants. **A** - Immunoblots obtained by modified PhosTag™ SDS-PAGE approach using the antibody against Lhcb2 protein. The upper band corresponds to the phosphoprotein (P), lower band to the unphosphorylated protein (NP). **B**, **C** – Changes in phosphorylation of Lhcb1 (**B**) and Lhcb2 (**C**) proteins in runner bean plants, revealed by Western blot of modified PhosTag™ SDS-PAGE gels. Changes were calculated as P/(P + NP) and normalized to 0% at time point 0 h. Data are mean values ± SD from three to five independent experiments. For control conditions plants were kept in darkness at 21 °C for three days (dark) or in a growth chamber in day/night cycle conditions (light). Results marked with hash, dollar, and ampersand differ significantly at *p* = 0.05 (one-way ANOVA with post hoc Tukey test) between dark-chilling vs. light, dark-chilling vs. dark, and light vs. dark conditions, respectively

This work focuses on deciphering the biochemical mechanism of the dark-chilling-induced increase in LHCII phosphorylation observed in runner bean. We started with detailed analysis of changes in phosphorylation of two major LHCII proteins: Lhcb1 and Lhcb2, to establish the kinetics of this process using a two-layer PhosTag™ SDS PAGE (Fig. 1). The advantage of this approach is the slower migration of phosphorylated proteins during electrophoresis, which results in the separation of two protein pools, visible as separate bands: phosphoproteins and unphosphorylated proteins on the same Western blot [e.g. 42], (Fig. 1A). Under dark-chilling conditions in runner bean, a vast increase in phosphorylation of both Lhcb1 (Fig. 1B) and Lhcb2 (Fig. 1C) proteins was observed. The maximum level of phosphorylation was reached after 9 h of dark-chilling for both proteins, and after 24 h returned to the level from the beginning of the experiment (0 h) (Fig. 1B, C). Lhcb2 reached a higher level of phosphorylation (37%), when compared to Lhcb1 (28%), however, the difference in phosphorylation levels of those two proteins on light is significantly higher (22% and 46%, respectively) as was also reported before [42, 56]. At the end of the night period in our experimental model (0 h), 9.6 ± 5.6% of Lhcb1 and 13.6 ± 8.3% of Lhcb2 proteins were phosphorylated in the runner bean’s thylakoids. These values represents the phosphorylation level without normalization described in Materials and methods section. Because most changes in LHCII phosphorylation were observed during the first 24 h of dark-chilling, we narrowed down the time frame to 24 h.

### Phosphorylated LHCII associates with PSI-LHCI complex in grana and stroma lamellae fractions under dark-chilling

In the next step, we evaluated if dark phosphorylated LHCII builds the PSI-LHCI-LHCII complex as observed during light-induced state transitions.

**Fig. 2:**
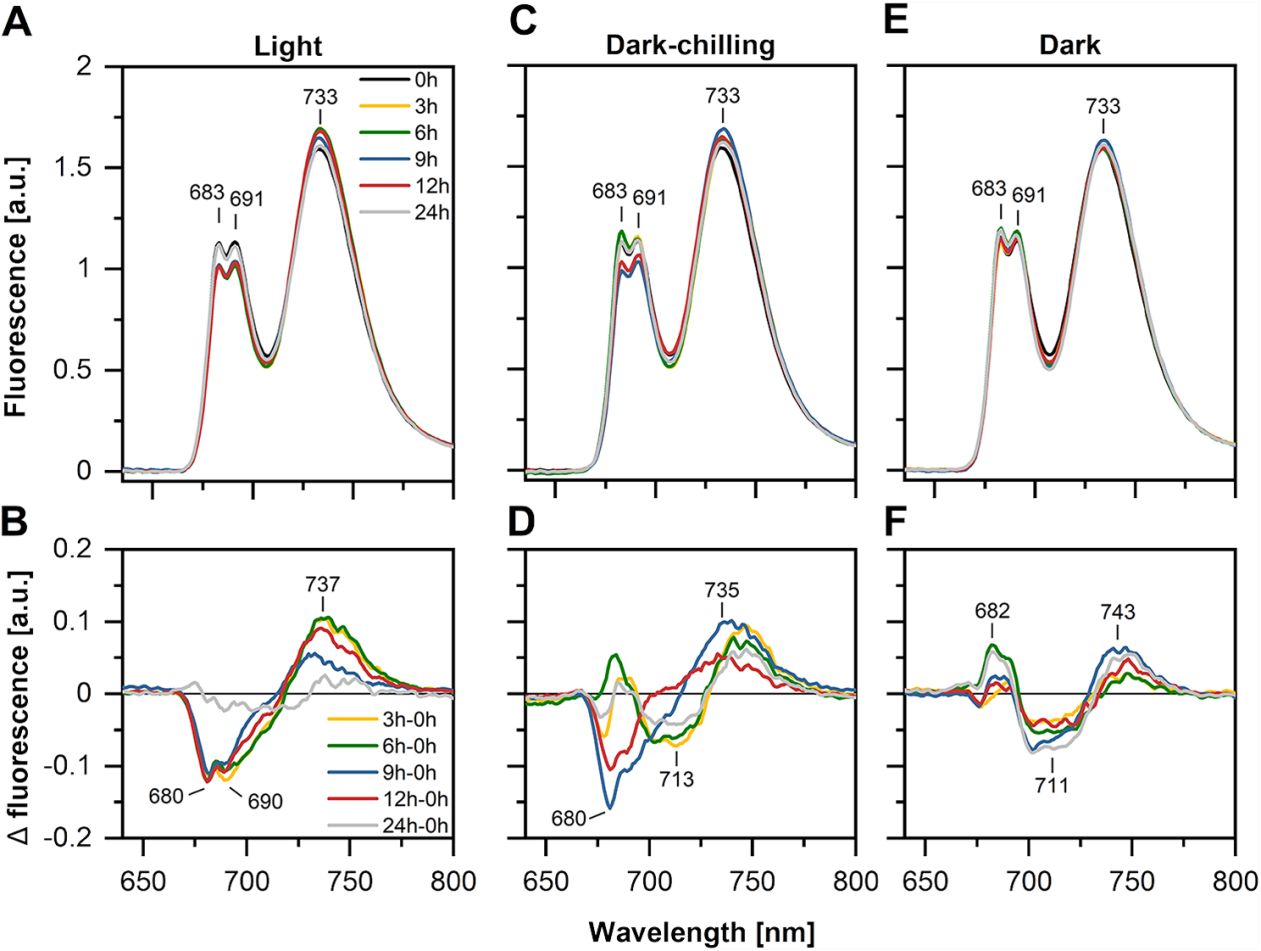
PSI fluorescence increases during dark-chilling of runner bean plants. Fluorescence emission spectra (Ex 440 nm) recorded at 77 K of thylakoids isolated from leaves of runner bean plants from light (**A**), dark-chilling (**C**) and dark conditions (**E**). Lower panels present the corresponding difference spectra (time-point minus time 0 h) for light (**B**) dark-chilling (**D**) and dark conditions (**F**). The spectra (**A**, **C**, **E**) were normalized to the same area under the spectrum (100 arbitrary units), and the arithmetic differences (**B**, **D**, **F**) between them were calculated. The presented spectra are the mean values of three independent experiments

Low-temperature fluorescence spectra (Fig. 2) of control light conditions samples showed a decreased contribution of LHCII trimers (a negative band at a difference spectra with a maximum at 680 nm) and an increased contribution of PSI (a positive band at a difference spectra with a maximum shifted to around 735 nm) already after 3 h of illumination and maintained on light. Similar observation was detected in dark-chilling samples after 9 and 12 h of dark-chilling (Fig. 2C, D). This effect is commonly attributed to the increased PSI antenna cross-section due to the P-LHCII trimer association [2]. In contrast, under dark conditions in control temperature we did not observe the decrease in the signal associated with LHCII-PSII complex (Fig. 2E, F).

**Fig. 3:**
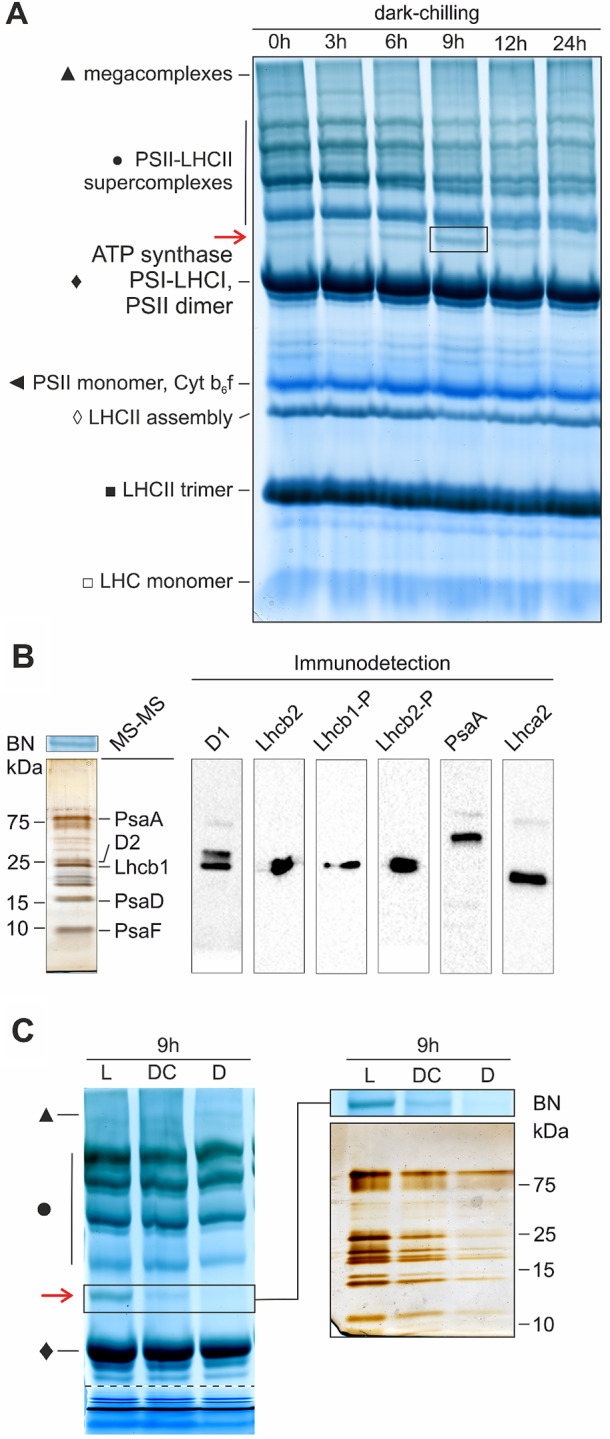
PSI-LHCI-LHCII complex is formed during dark-chilling of runner bean plants. **A** – Blue Native PAGE of thylakoids isolated from runner bean plants exposed to 0–24 h of dark-chilling and solubilized with 1% β-dodecylmaltoside. The appearing PSI-LHCI-LHCII supercomplex is marked by an arrow. **B** – Analysis of the PSI-LHCI-LHCII band composition. The band from 9 h of dark-chilling (marked with a black frame) was cut out of BN gel, denatured, and separated by SDS-PAGE. Proteins were identified by mass spectrometry (full list in Table S3) and immunodetection. **C** – Comparison of the protein composition of BN gel area related to PSI-LHCI-LHCII in runner bean thylakoids isolated after 9 h of all experimental treatments (the image of BN gel below dashed line was squeezed to fit the page). The presented gels and blots are representative of two independent experiments

To confirm the presence of PSI-LHCI-LHCII complex in dark-chilled runner bean we performed Blue Native electrophoresis. We observed no significant changes in the number of supercomplexes, PSII dimers, or LHCII content (Fig. S1). However, we observed the increase of abundance of a band, located just below the PSII-LHCII supercomplexes section, under dark-chilling and light control, but not under dark conditions (Fig. 3A, Fig. S1). The new supercomplex was most abundant after 3 h of light control and 9 h of dark-chilling (Fig. S1). Detailed analysis of the new band’s composition was performed using two-dimensional electrophoresis, Western-blot, and mass spectrometry (Fig. 3B, Table S3). We confirmed that it is composed not only of PSI proteins: PsaA, PsaD, PsaF, Lhca2, but also of PSII antennae: Lhcb1 and Lhcb2, both in phosphorylated form (Fig. 3B). These results show that dark-chilling induces the formation of the PSI-LHCI-LHCII complex in runner bean, confirmed both by DDM and digitonin digestion of thylakoids membranes (Fig. 3, S2). Comparative two-dimensional electrophoresis of the BN gel region corresponding to PSI-LHCI-LHCII position for all experimental variants after 9 h of the experiment confirmed its presence in runner bean under dark-chilling and light control conditions only. Moreover, the protein composition of the detected supercomplex was identical indicating the same organization in light and after dark chilling (Fig. 3C).

**Fig. 4:**
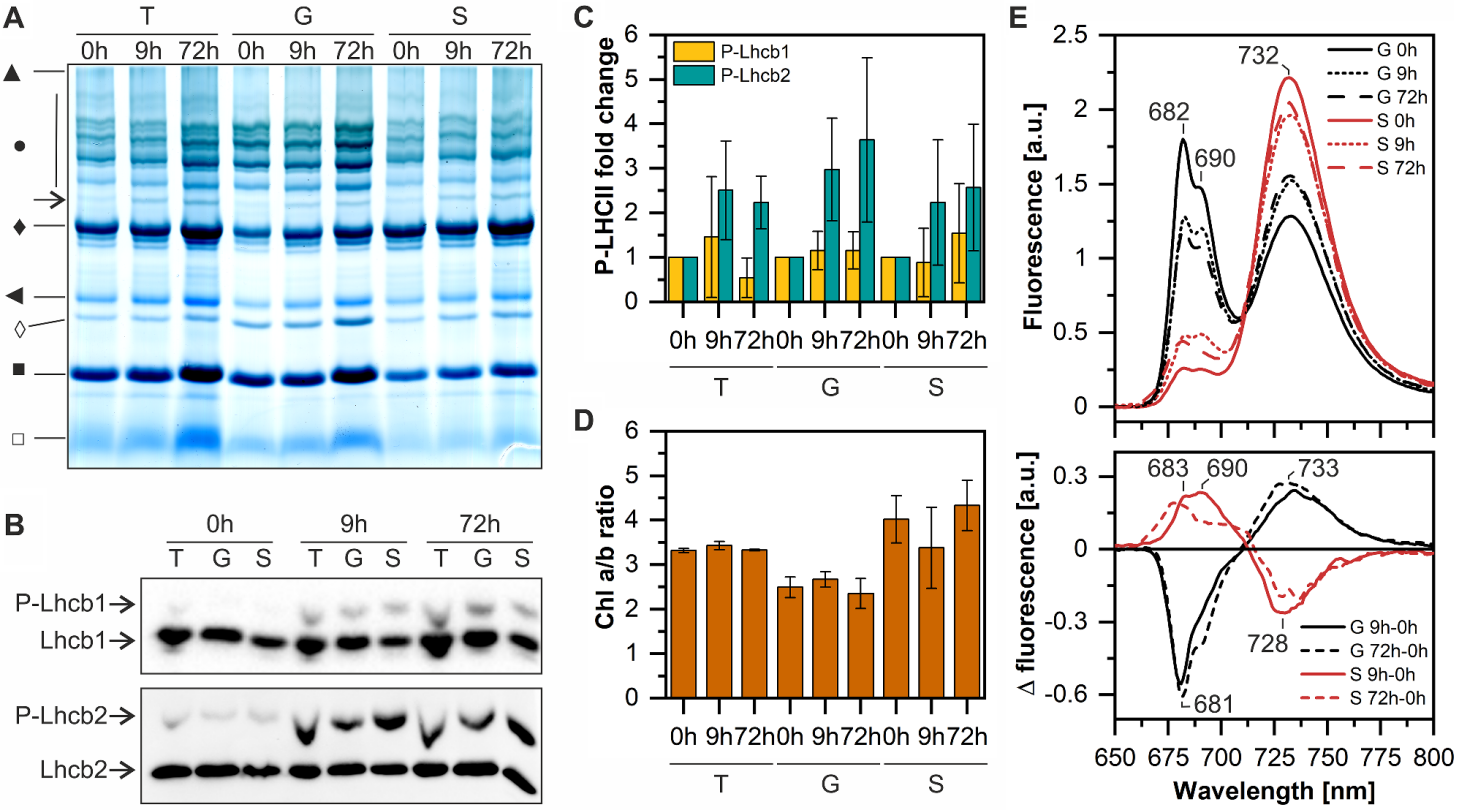
PSI-LHCI-LHCII complex locates in grana and stroma lamellae fractions of runner bean’s thylakoids during dark-chilling. **A** – Blue Native PAGE of thylakoids (T) and thylakoid fractions: grana (G) and stroma (S) lamellae isolated from runner bean plants exposed to 0, 9, and 72 h of dark-chilling. The arrow indicates the PSI-LHCI-LHCII complex. The legend for other symbols: ▲, megacomplexes; ●, PSII-LHCII supercomplexes; ♦, ATP synthase, PSI-LHCI, PSII dimer; ◄, PSII monomer, Cyt b_6_f; ◊, LHCII assembly; ■, LHCII trimer; □, LHCII monomer. **B** – Immunoblots obtained by modified PhosTag™ SDS-PAGE approach using antibodies against Lhcb1 and Lhcb2 proteins. **C** – Changes in Lhcb1 and Lhcb2 phosphorylation in isolated thylakoid fractions; data were normalized to the 0 h. **D** – Chl a/b ratios in thylakoids, grana, and stroma lamellae preparations. **E** – Upper panel – Low-temperature fluorescence spectra of thylakoids and thylakoid fractions isolated from runner bean plants exposed to 0, 9, and 72 h of dark-chilling. Lower panel – Comparison of the normalized fluorescence emission difference spectra (time-point minus time 0 h) for the runner bean thylakoids and thylakoid fractions (Ex 440 nm). The presented gels, blots, and spectra are representative of at least three independent experiments; data are mean values ± SD

We further assessed the lateral location of the newly formed PSI-LHCI-LHCII supercomplex in the thylakoid membranes, by performing digitonin fractionation of total thylakoids followed by electrophoretic and spectroscopic analysis of obtained grana and stroma lamellae thylakoid fractions (Fig. 4). We have selected two experimental time points: (i) 9 h, when the PSI-LHCI-LHCII supercomplex was the most abundant, and (ii) 72 h, the last time point of the experiment, to verify whether the complex is still present in the thylakoid membrane. Obtained fractions’ purity was confirmed by SDS PAGE and Western blot (Fig. S3). PSI-LHCI-LHCII supercomplex was present in grana and stroma lamellae fractions after 9 h and 72 h of dark-chilling (Fig. 4A). PSI-LHCI-LHCII presence was consistent with the presence of phosphorylated Lhcb1 and Lhcb2 registered in fractionated samples (Fig. 4B, C). Note that in Fig. 1 LHCII protein phosphorylation after 72 h of dark-chilling was significantly lower than after 9 h time-point (Fig. 1). Although plants were growing in controlled conditions observed discrepancy in phosphorylation status at 72 h time point strongly correlates with the season of cultivation; i.e., only plants grown outside the natural vegetative period of beans (late fall to spring) were characterized by high phosphorylation at 72 h of dark-chilling. Latest studies indicate that seasonal internal clock of plants can influence their growth [57], however, in the context of LHCII protein phosphorylation status it requires further investigation.

The lower Chl *a*/*b* ratio in stroma lamellae fraction after 9 h of dark-chilling (Fig. 4D) suggested the enrichment of stroma lamellae with Chl *b*-containing complexes – most likely LHCII. Low-temperature fluorescence spectra of grana fraction showed the decreased contribution of LHCII trimers (680 nm) and increased contribution of LHCI (735 nm) (Fig. 4E), similar to intact runner bean’s thylakoids (Fig. 2B). An opposite effect was observed for stroma lamellae, where the contribution of LHCII trimers increased and the contribution of LHCI decreased (Fig. 4E).

### Photosynthetic complexes stoichiometry under dark-chilling

Spectroscopic analysis of total thylakoid fractions showed that chlorophyll *a*/*b* ratio in runner bean was higher after 9 h of dark-chilling (Table 1). However, this effect was only temporary – after 72 h of dark-chilling, Chl *a*/*b* ratio returned to the start point value (Table 1). No significant changes in PSII content were observed for runner bean from dark and dark-chilling conditions. PSI quantification showed a reduction in functional P700 content after 72 h of dark-chilling and dark in runner bean (Table 1).

**Table 1:** Changes in the photosynthetic complexes stoichiometry in runner bean under dark-chilling stress. Average values ± SD of chlorophyll *a*/*b* ratio and photosynthetic complexes content per mole of chlorophyll of runner bean plants after 9 and 72 h of dark (D) and dark-chilling (DC)

Sample	Chlorophyll *a*/*b*	Cytochrome b_6_f [mmol×mol Chl^− 1^]	PSII [mmol×mol Chl^− 1^]	P700 [mmol×mol Chl^− 1^]
0 h	3.32 ± 0.05	0.96 ± 0.10	1.80 ± 0.21	1.75 ± 0.13
9 h DC	3.43 ± 0.09*	0.94 ± 0.07	1.85 ± 0.18	1.58 ± 0.15
72 h DC	3.33 ± 0.02	0.90 ± 0.06	1.68 ± 0.08	0.90 ± 0.08*
9 h D	3.39 ± 0.05	1.03 ± 0.07	2.03 ± 0.18	1.86 ± 0.15
72 h D	3.25 ± 0.02	0.94 ± 0.09	1.93 ± 0.19	1.30 ± 0.23*

Values denoted with an asterisk are significantly different from 0 h at *p* = 0.05 (one-way ANOVA with post hoc Tukey test, *n* = 6–8)

### STN7 kinase activation under dark-chilling conditions

**Fig. 5:**
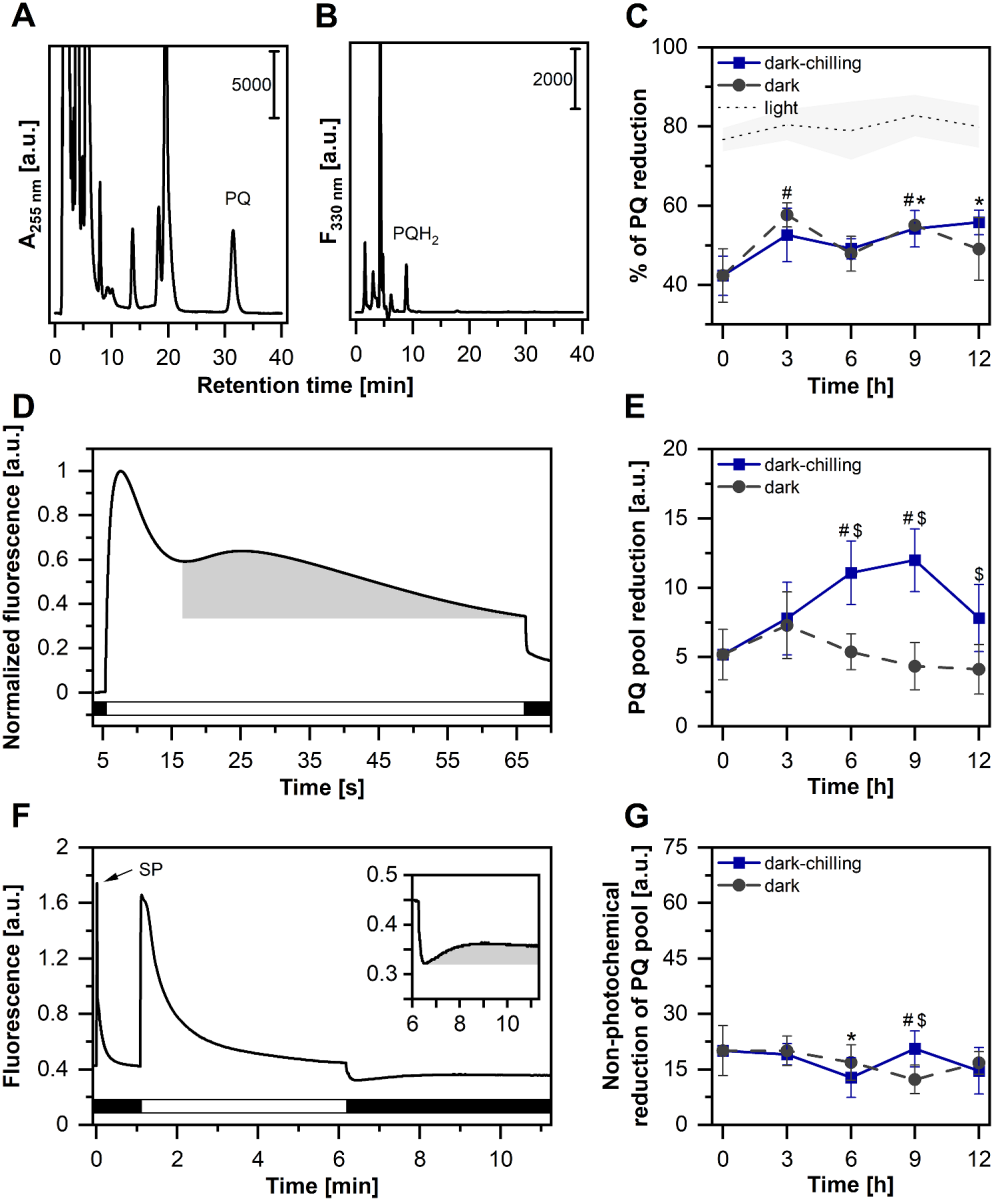
Plastoquinone pool reduction increases in runner bean under dark-chilling conditions. **A**, **B** – The analysis of the plastoquinone (PQ) and plastoquinol (PQH_2_) content in ethyl acetate extracts from runner bean leaves. Exemplary absorption (**A**) and fluorescence (**B**) chromatograms are shown. **C** – changes in PQ reduction in runner bean during dark-chilling and dark conditions. A black dashed line with gray shade indicates the level of PQ reduction (mean values ± SD) after exposition to the white light of 1000 µmol photons m^–2^ s^–1^ intensity for 30 s, i.e., conditions allowing total reduction of photoactive PQ pool. **D** – Analysis of slow chlorophyll *a* in vivo fluorescence kinetics in runner bean plants. Curves are normalized to the maximal value. **E** – Changes in PQ pool reduction (calculated as the area under the curve showed as a gray shade in panel **D**) in bean during dark-chilling and dark conditions. **F** – Analysis of non-photochemical reduction of the PQ pool using chlorophyll a in vivo fluorescence in runner bean plants. An increase in signal in the dark period indicates a non-photochemical reduction of PQ. **G** – Changes in non-photochemical reduction of the PQ pool (calculated as the area under the curve showed as a gray shade in panel **F**) in runner bean during dark-chilling and dark conditions. White and black bars on panels **D** and **F** represent the illumination and dark phases of the measurements, respectively. SP, saturating light pulse. The data are mean values ± SD of 3–4 (panel **C**), 5–11 (panel **E**), and 7–24 (panel **G**) replicates from two independent experiments. Results marked with an asterisk (dark-chilling) and hash (dark) differ significantly from time-point 0 h at *p* = 0.05 (one-way ANOVA with post hoc Tukey test), while these marked with dollar differ significantly between dark-chilling vs. dark within single time point

**Fig. 6:**
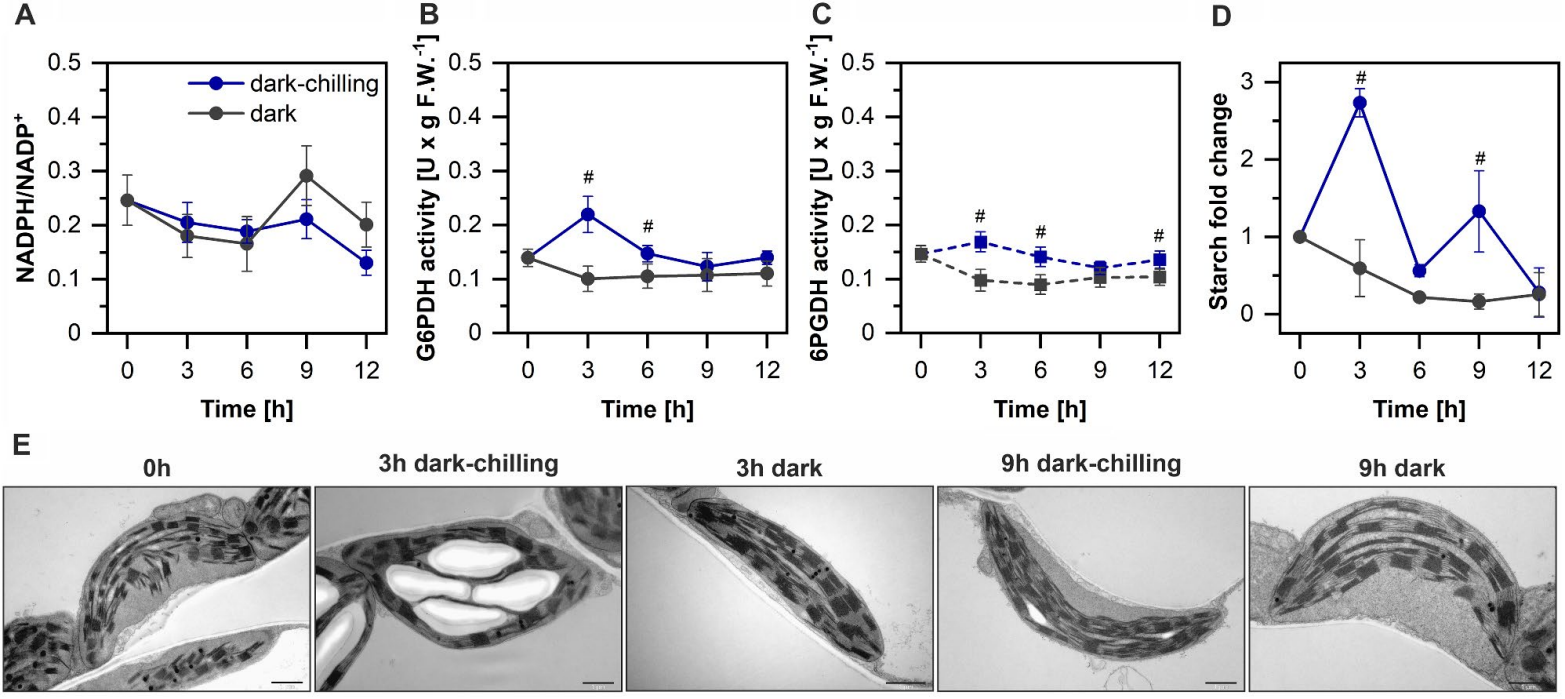
Stress-correlated activation of metabolic processes in runner bean during dark-chilling. **A** – Changes in NADPH/NADP^+^ ratio in runner bean leaves during dark-chilling and dark conditions. Nucleotide concentration was calculated in nmol × g F.W.^−1^. **B**-**C** – Changes in the activity of glucose-6-phosphate dehydrogenase (**B**) and 6-phosphogluconate dehydrogenase (**C**) in runner bean leaves during dark-chilling and dark conditions. **D** – Changes in the starch content in runner bean during dark-chilling and dark conditions. Starch concentration was calculated in µmol glucose × g F.W.^−1^. **E** – Exemplary TEM images of mesophyll cell chloroplasts of runner bean from selected time points of dark-chilling and dark conditions. The data are mean values ± SD of 3–5 replicates from three independent experiments; results marked with hash differ significantly at *p* = 0.05 (one-way ANOVA with post hoc Tukey test) between dark-chilling vs. dark. TEM images are representative of two separate experiments. U – µmol NADPH × min^− 1^; F.W. – fresh weight

To establish the pathway of STN7 kinase activation under dark-chilling conditions, we performed several complementary spectroscopic and biochemical analyses (Figs. 5 and 6). Since STN7 activation on light requires a reduced PQ pool, in the first step we analyzed whether such conditions i.e. increase in PQ pool reduction, are also present in dark-chilled runner bean plants.

We started with the analysis of the total leaf PQ and PQH_2_ contents by means of HPLC (Fig. 5A-C). In runner bean, we showed a slight, almost linear, increase of PQ reduction from 40 to 50% of the total PQ pool during 12 h of dark-chilling conditions (Fig. 5C). Similar pattern was also observed in dark conditions (Fig. 5C). The PQ reduction in the dark-chilling and dark conditions was between 60 and 75% of the maximal light-induced level (Fig. 5C).

Further, we measured the slow kinetics of Chl *a* fluorescence to verify changes in the reduction status of the photoactive PQ pool, in runner bean leaves under dark-chilling and dark conditions (Fig. 5D-E, S5A-B). This is since only the photoactive PQ pool can activate STN7. Slow kinetic fluorescence traces analysis revealed that in runner bean the reduced PQ pool increased gradually up to 9 h of dark-chilling (Fig. 5E). In contrast such an effect was not observed in dark conditions (Fig. 5E). The highest noted value of reduced PQ pool in runner bean was in line with the peak level of LHCII phosphorylation, observed after 9 h of dark-chilling (Fig. 1B, C). To reveal whether the increase of the reduction of PQ pool in the dark-chilled runner bean is directly related to the dark-operating NDH and/or ferredoxin: quinone reductase (FQR) enzymes [46], we measured the nonphotochemical reduction of the PQ pool using in vivo chlorophyll fluorescence approach (Fig. 5F-G, S5C-D). Since the non-photochemical reduction of PQ is possible under dark-chilling and dark conditions (Fig. 5G), results suggest that in runner bean the activities of FQR and/or NDH were present under both experimental conditions.

FQR and NDH activities depend (directly or indirectly) on the availability of reduced NADPH, thus to establish further steps of STN7 activation in the darkness we tested NADPH and NADP^+^ contents (Fig. 6A). We observed a quite steady NADPH/NADP^+^ ratio during 9 h of dark-chilling with slight decrease after 12 h of dark-chilling. In dark conditions the NADPH/NADP^+^ ratio is similar to those in dark-chilling except the 9 h where transient increase was observed (Fig. 6A). The lack of expected NADPH accumulation in runner bean under dark-chilling might be caused by the instant usage of this reducing force.

It is well known that NADPH in the chloroplast in the dark is produced by OPPP glucose-6-phosphate dehydrogenase (G6PDH) and 6-phosphogluconate dehydrogenase (6PGDH) enzymes [58] and their substrates can be supplied from starch degradation pathway [59]. Therefore, we showed that in runner bean there was a transient increase in the activities of both G6PDH and 6PGDH after 3 h of dark-chilling (Fig. 6B, C). Further, we detected an almost 3-fold increase in starch content in runner bean leaves after 3 h of dark-chilling, with the second smaller peak after 9 h of treatment (Fig. 6D). On the contrary, a constant decrease in starch content in dark-treated runner bean was observed (Fig. 6D). Such biochemical observations were confirmed by electron microscopy analysis in which starch accumulation was assessed qualitatively (Fig. 6E).

**Fig. 7:**
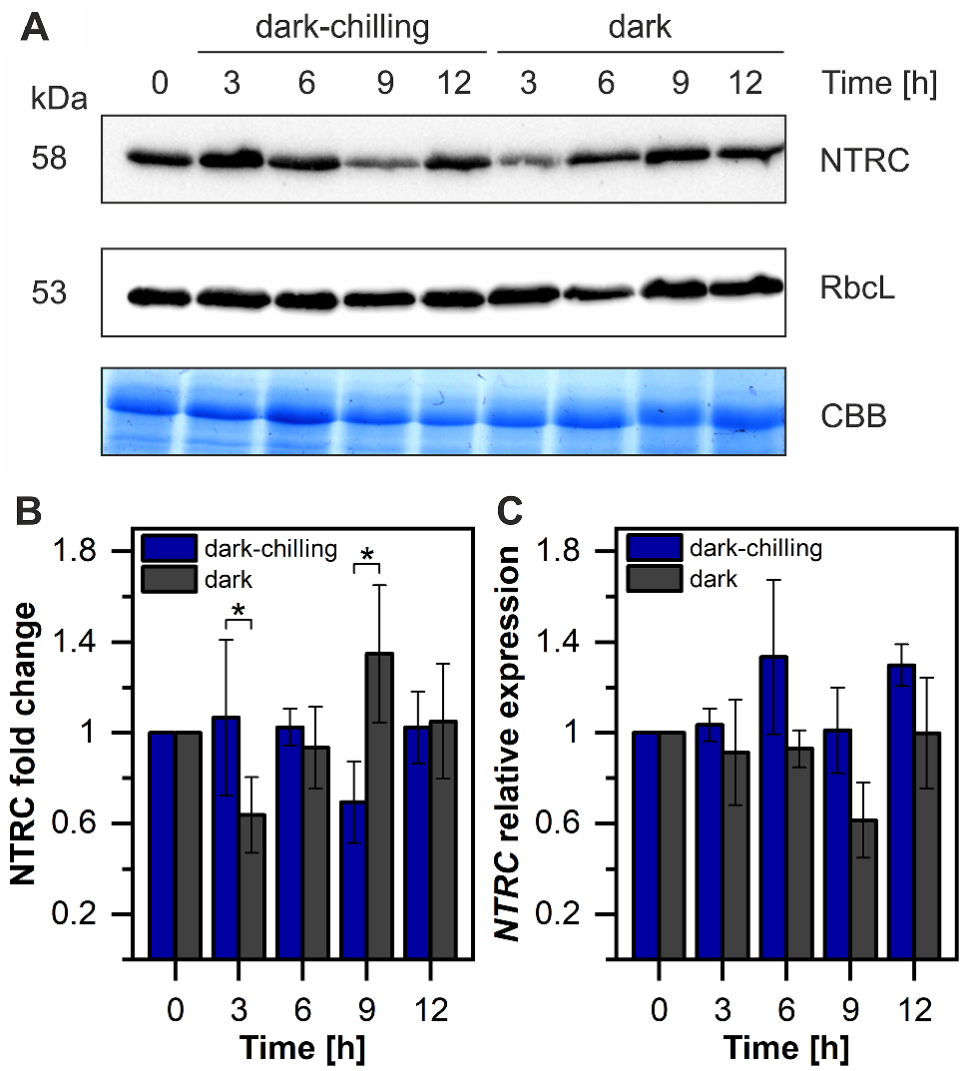
NTRC protein level fluctuates under dark-chilling and dark conditions in runner bean plants. **A** – Immunoblots showing levels of NTRC and large subunit of Rubisco (RbcL) proteins in 10 µg of total protein extract isolated from leaves of runner bean plants. Coomassie Brilliant Blue staining (CBB) was used as a sample loading control. The presented blots are representative of three independent experiments. **B** – Changes in the NTRC protein levels in runner bean leaves exposed to dark-chilling (blue) and dark (grey) conditions, revealed by Western blot. Results were normalized to the amount of RbcL and then to 1 at time point 0 h. Data are mean values ± SD from three independent experiments. **C** – Changes in the *NTRC* relative expression. Data are mean values ± SE from four independent experiments. Results marked with a star differ significantly at *p* = 0.05 (one-way ANOVA with post hoc Tukey test) between dark-chilling vs. dark

Our next question regarded the elucidation of the dark-chilling induced starch accumulation pathway as the next detected important upstream step in STN7 kinase activation. Since Pérez-Ruiz et al. [31] showed that the NTRC enzyme is operating under dark conditions and activates both ADP-glucose pyrophosphorylase (AGPase), a key starch synthesis enzyme [60], and NDH, a source of nonphotochemically reduced PQ [32], we decided to test NTRC protein and transcript levels in our experimental setup (Fig. 7).

Under dark-chilling of runner bean plants the NTRC protein level was stable during 12 h with a transient drop after 9 h (Fig. 7B). On the contrary, dark conditions induced more visible fluctuations of NTRC level, with a decrease after 3 h and an increase after 9 h (Fig. 7B). Expression analysis of *NTRC* transcript showed some fluctuations during 12 h under both experimental conditions, however without significant differences (Fig. 7C). We suspect that the lack of NTRC protein level decrease in dark-chilling compared with dark conditions at 3 h of the experimental treatment might be responsible for observed starch accumulation in the runner bean.

## Discussion

Low temperature is one of the common stress factors which affects plant growth, especially in the temperate climate zone, where during the spring season high daily fluctuations of temperature are observed. Such conditions activate a plethora of recognized as well as not yet identified metabolic responses. However, the exact biochemical mechanisms, even for already described responses, are not fully understood.

In this work, we decipher the mechanism of dark-chilling-induced LHCII phosphorylation, the phenomenon which we reported in our previous study [15, 20]. Moreover, we indicate the implications of P-LHCII level on the supramolecular organization of photosynthetic complexes. We started with tracking the time resolved phosphorylation pattern in the runner bean plants, and showed that the LHCII phosphorylation level increases up to 35% of the total LHCII fraction after 9 h of dark-chilling conditions. This value is comparable to the one obtained in light conditions for the studied species (Fig. 1).

### Role of LHCII phosphorylation in a reorganization of CP complexes and thylakoid membrane spatial structure under dark-chilling

LHCII phosphorylation, usually related to light-induced state transition, causes the reorganization of photosynthetic complexes [reviewed in 61]. We observed the formation of state 2 characteristic PSI-LHCI-LHCII complex in runner bean in light and under dark-chilling conditions (Fig. 3). Detailed membrane fractionation analysis demonstrated the presence of P-LHCII and PSI-LHCI-LHCII complex both in grana and stroma lamella fractions isolated from dark-chilled runner bean leaves (Fig. 4), indicating that not all P-LHCII are mobile in terms of long-range grana – stroma lamellae distance. This is in line with recent research, postulating the presence of two spatially separated pools of LHCII with limited mobility [11, 12]. In stroma lamellae fraction isolated from dark-chilling, we observed the increase of 77 K fluorescence signal below 700 nm (Fig. 4E), a slight decrease in Chl *a*/*b* ratio (Fig. 4D), and an increase of total LHCII content (Fig. S4). These suggest the migration of a fraction of P-LHCII pool outside grana membranes. Interestingly, there is no evidence for partial unstacking of grana marginal regions under dark-chilling (Fig. S6), postulated as a necessary step of LHCII migration outside the grana [62, 63]. It means, that the P-LHCII relocation to the stroma lamellae can be achieved without major membrane reconfigurations as shown by Verhoeven et al. using the single chloroplast *in folio* fluorescence imaging [64]. Therefore, we speculate that the magnitude of P-LHCII grana to stroma migration might depend on the species-specific organization of grana stacks in which the ratio of end- to stacked membranes can vary significantly [65]

### Pathway leading to LHCII phosphorylation under dark-chilling

**Fig. 8:**
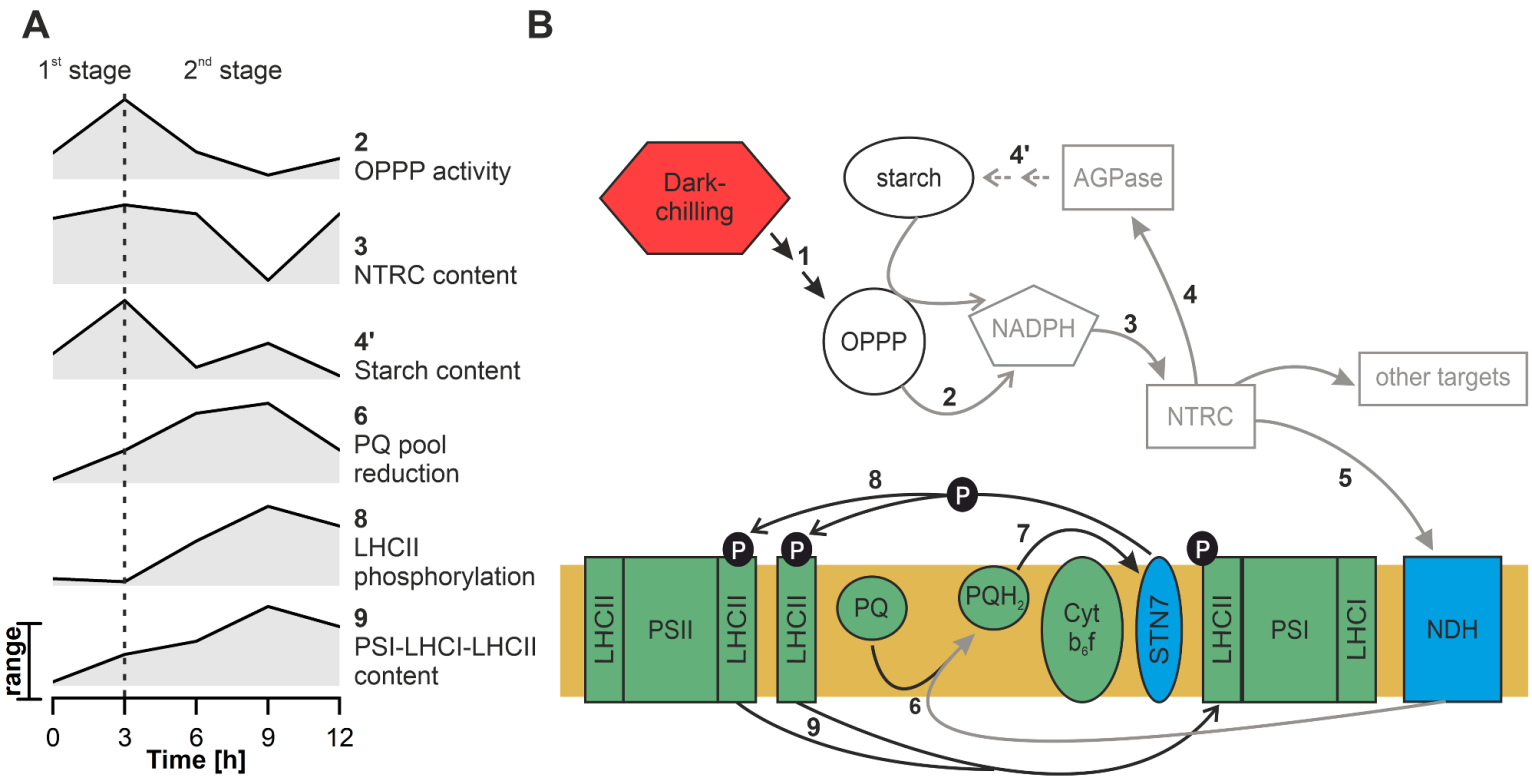
Proposed pathway of the light-independent STN7 kinase activation in runner bean plants in response to dark-chilling. **A** – Graphs showing the magnitude of selected metabolic processes presented on the time scale of dark-chilling exposure of runner bean. Data were taken from Figs. 1, 3 and 5–7 and normalized to 1 and 0 at maximal and minimal values, respectively. **B** – Scheme of the pathway leading to the light-independent activation of STN7 kinase and LHCII phosphorylation in runner bean; numbers show the order of events. The proposed pathway starts with dark-chilling induced (1) activation of OPPP enzymes (2). NADPH produced by OPPP enzymes activates NTRC (3), which then triggers AGPase (4), resulting in starch synthesis (4’). The subsequent target of NTRC is thylakoid membrane-located NDH (5), which activity transfers electrons to the PQ pool, causing its reduction (6) – this process is delayed compared to starch synthesis. Increased reduction of the PQ pool triggers STN7 kinase (7), resulting in LHCII phosphorylation (8) and finally, state transitions (9). Note that not all of proposed pathway elements were confirmed experimentally. Elements of the scheme showed in gray represent speculative parts of the proposed mechanism

In control light conditions the LHCII phosphorylation in runner bean reaches maximal level after 6 h of illumination (Fig. 1). Under dark-chilling this process is much slower, starts between 3rd and 6th h, and reaches a maximum after 9 h (Fig. 1). Therefore, it can be presumed that the pathway activating STN7 kinase in dark-chilling conditions contains additional steps as compared with light activation. In this work we focused on deciphering the steps of this process, revealing the putative mechanism of dark-chilling-induced LHCII phosphorylation. The proposed model of the STN7 kinase activation mechanism in runner bean under dark-chilling conditions based on the results provided in this work is summed up in Fig. 8.

In detail, after 3 h of dark-chilling, a significant short-lasting increase in the activities of two OPPP enzymes: G6PDH and 6PGDH was observed (Fig. 6B, C). The activity of OPPP enzymes is a well-known source of NADPH in the dark [58]. NADPH generated in the OPPP was shown to reduce, i.e. activate, NTRC in the dark [31, 32]. Despite observed OPPP enzyme activation, we did not register significant NADPH accumulation suggesting increased NADPH usage in dark-chilled samples (Fig. 6A-C). However, we analyzed the NTRC transcript and protein levels as well as the downstream effects of NTRC activity. At three hours of dark-chilling we detected significantly higher NTRC protein level comparing with dark conditions (Fig. 7B). Simultaneously, we observed the NTRC activity manifestation in runner bean as the induction of starch synthesis, which occurred after 3 and 9 h of dark-chilling (Fig. 6D). Dark-activation of AGPase, a key enzyme of the starch biosynthesis pathway, was demonstrated to be executed in the dark by NTRC [60, 66]. The increased AGPase activity and starch accumulation in response to chilling stress was observed in the maize line [67] and, similarly to our experiments, the starch formation was detected also in tomato [68] and cucumber cotyledons [69] under chilling and dark-chilling conditions, respectively. Starch synthesis occurring in runner bean in dark-chilling conditions was confirmed by two independent methods – TEM and biochemical assay (Fig. 6D, E). However, the source of sugars necessary for starch synthesis in the dark-chilling conditions remains to be discovered.

Since the NTRC activity was shown to enhance the NDH-dependent cyclic electron flow which increases the reduced PQ pool [32], we also investigated the non-photochemical reduction of PQ. Since cyclic electron flow mediated by NDH was shown to be enhanced by cold stress [70], the NDH pathway probably has a predominant role in the nonphotochemical reduction of PQ in the dark-chilling.

In runner bean, we detected the gradual increase in reduced PQ pool with the maximum after 9 h of dark-chilling (Fig. 5E). In this time point, the significant increases in nonphotochemically reduced PQ pool (Fig. 5G) and LHCII phosphorylation (Fig. 1) were detected. The enhanced reduction of the PQ pool in stress conditions was already noted for rice growing under light-chilling [71]. However, our results indicate that this effect is also present under dark-chilling when the PSII-driven PQ reduction is inactive. Therefore, the nonphotochemical reduction of PQ, resulting from the activation of metabolic pathways in the chloroplast stroma in which NTRC plays a pivotal role, might be the final step triggering STN7 kinase activation in runner bean under dark-chilling conditions.

Summarizing, we distinguish two proposed stages of studied mechanism. First proposed stage includes OPPP activation (Fig. 8B, arrows 1-2), possible NTRC and AGPase activation (Fig. 8B, arrows 3-4) and confirmed starch accumulation (Fig. 8B, arrow 4’). This stage occurs in the stroma of chloroplasts and is associated with diffusion in the aqueous solution. It is completed within the first three hours of dark-chilling (Fig. 8A). The proposed second stage occurs inside the thylakoid membrane. It is composed of possible NDH activation (Fig. 8B, arrow 5), PQ pool reduction (Fig. 8B, arrow 6), STN7 kinase activation (Fig. 8B, arrow 7), and finally LHCII phosphorylation (Fig. 8B, arrow 8). The second stage takes place between 3rd and 9th hour (Fig. 8A) of dark-chilling. These lipid bilayer-located processes are slower, compared with stroma-located reactions indicating time separation of two proposed stages of studied mechanism (Fig. 8A).

Although in this work, we described the possible mechanism leading to the dark-chilling induced LHCII phosphorylation in detail taking into account also the time course of the following steps, the biological significance of this phenomenon remains puzzling. We can speculate on two competing hypotheses. In the first one, this process is considered an adaptive mechanism by which the runner bean mitigates the negative effects caused by low temperatures. For instance, by maintaining membrane stability in chilling conditions due to the presence of a highly mobile P-LHCII pool [72] and regulation of the cell turgor by starch synthesis/degradation [reviewed in 73] being a part of the studied phenomenon. The second hypothesis assumes that the observed mechanism is one of the metabolic components of runner bean chilling sensitivity by unnecessary activation of various processes (e.g., LHCII phosphorylation and starch accumulation) consuming ATP, that cannot be easily renewed under dark-chilling conditions. Since the biological role of observed effects could be considered from such two completely different perspectives, the clarification of the studied mechanism function requires further investigation also including other chilling sensitive and chilling tolerant species.

## Conclusions

In this study, we propose the light-independent STN7 kinase activation pathway which operates in runner bean under dark-chilling conditions. Obtained results suggest the direct connection between chloroplast thioredoxin system activity and reorganization of photosynthetic complexes, which sets a new perspective on the photosynthesis regulatory mechanisms able to operate in the absence of light. We showed that dark-chilling causes not only LHCII phosphorylation in runner bean, but also the formation of PSI-LHCI-LHCII complex, typical for light-induced state transitions. We linked the observed effect with the dark-chilling induced metabolic cascade possibly operating through the activation of OPPP, then NTRC-related both starch biosynthesis and nonphotochemical reduction of PQ. In consequence, photosynthetic complexes reorganization in runner bean in dark chilling conditions results from PQ pool-driven STN7 activation, indicating the possible connection between this chloroplast kinase activation and chilling stress response.

### Electronic supplementary material

Below is the link to the electronic supplementary material.

Supplementary Material 1

Supplementary Material 2

Supplementary Material 3

## Data Availability

The datasets used and/or analyzed during the current study are available from the corresponding author on reasonable request.

## Abbreviations

6PGDH: 6-phosphogluconate dehydrogenase
AGPase: ADP-glucose pyrophosphorylase
APS: Ammonium persulfate
BN: Blue-native
CP: Chlorophyll-protein
Cyt b_6_f: Cytochrome b_6_f complex
DDM: N-dodecyl-β-D-maltoside
FQR: Ferredoxin: quinone reductase
G6PDH: Glucose-6-phosphate dehydrogenase
LDS: Lithium dodecyl sulfate
LHCI: Light-harvesting complex I
LHCII: Light-harvesting complex II
NDH: NAD(P)H dehydrogenase-like complex
NTRC: NADPH-dependent thioredoxin reductase C
OE: Over-expressing
OPPP: Oxidative pentose phosphate pathway
PAR: Photosynthetically active radiation
PGR5: Proton Gradient Regulator 5 protein
P-Lhcb1: Phosphorylated Lhcb1
P-Lhcb2: Phosphorylated Lhcb2
P-LHCII: Phosphorylated light-harvesting complex II
PQ: Plastoquinone
PQH_2_: Reduced plastoquinone
PVDF: Polyvinylidene fluoride
ROS: Reactive oxygen species
STN7: State transition 7 kinase
TAP38/PPH1: Thylakoid-associated phosphatase 38/ protein phosphatase 1
TBS: Tris-buffered saline
TEMED: N, N,N’,N’-tetramethylethylenediamine
