# Supplementary figures and images for: Light-independent pathway of STN7 kinase activation under low temperature stress in runner bean (Phaseolus coccineus L.)
Source: BMC Plant Biol. 2024 Jun 7;24:513. doi: 10.1186/s12870-024-05169-3 (PMC11157908; doi:10.1186/s12870-024-05169-3)

**Fig 1A**

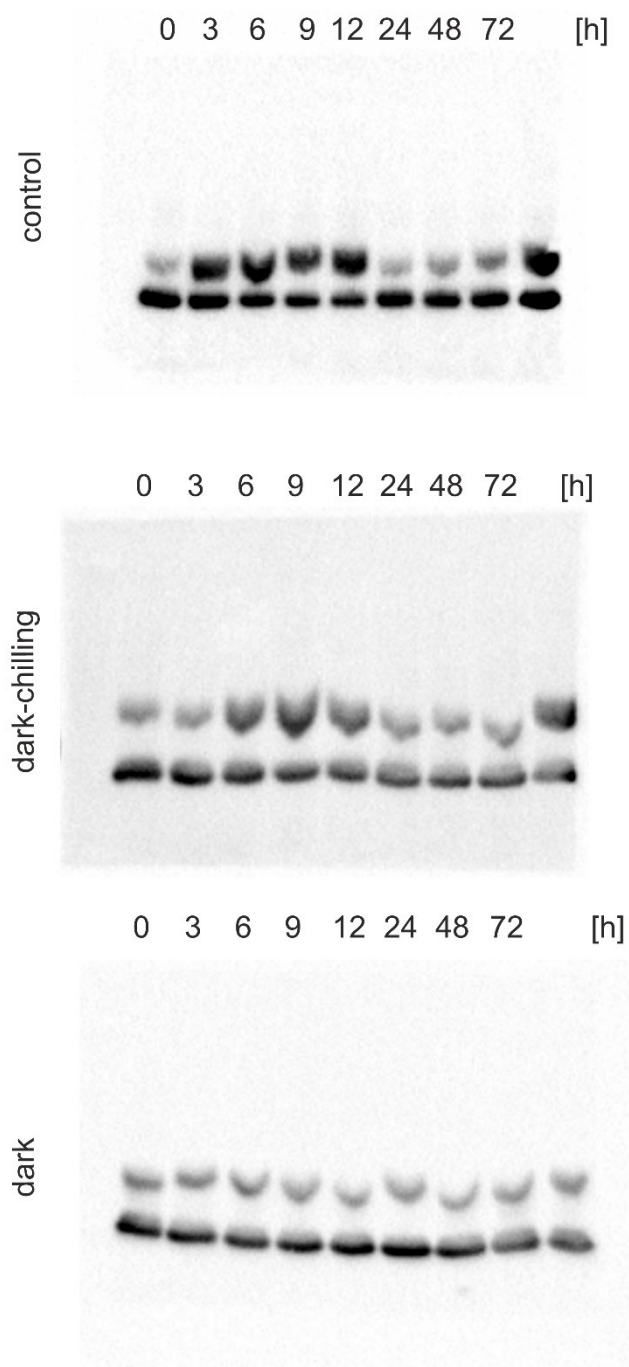

**Fig 3B**

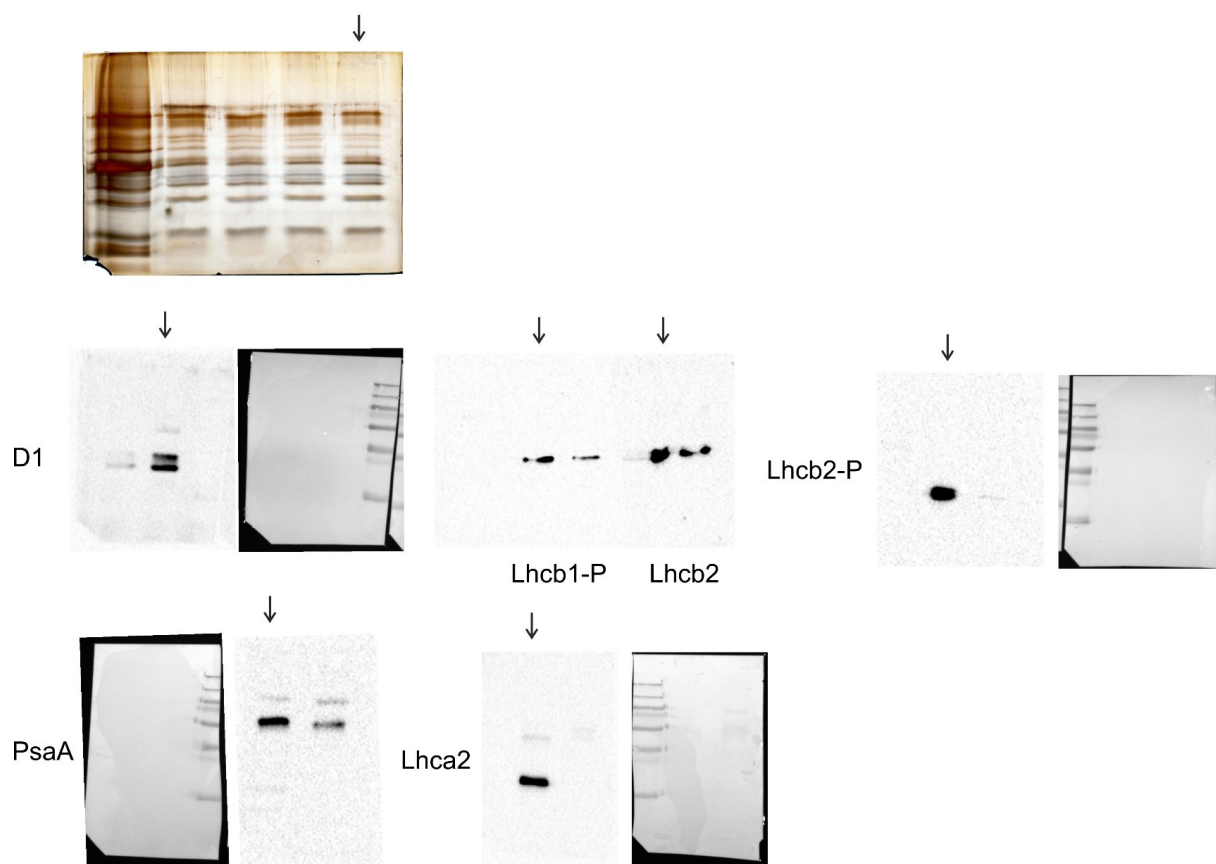

**Fig 3C**

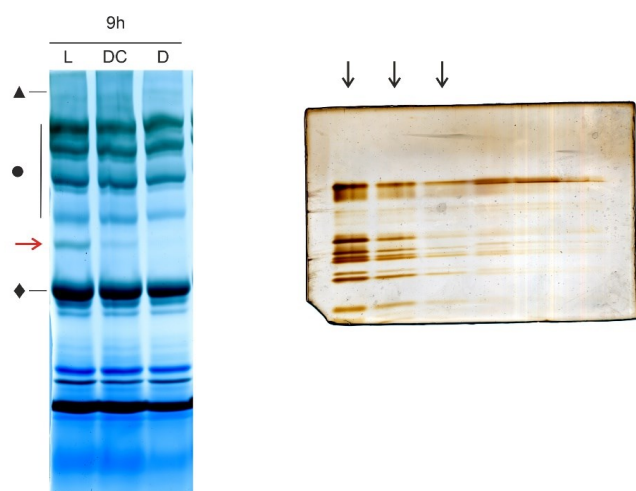

**Fig 4B**

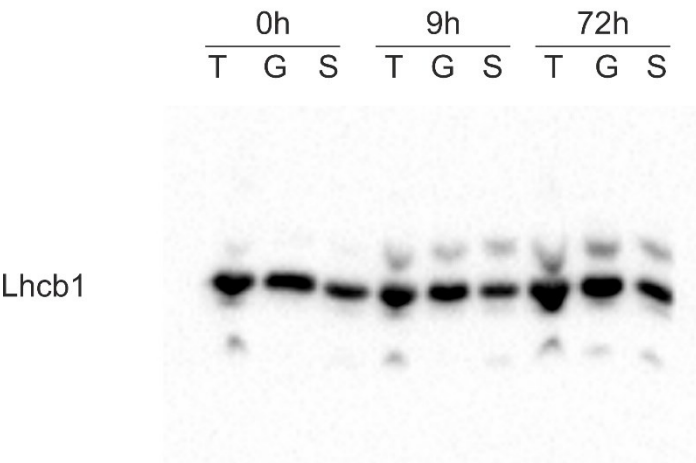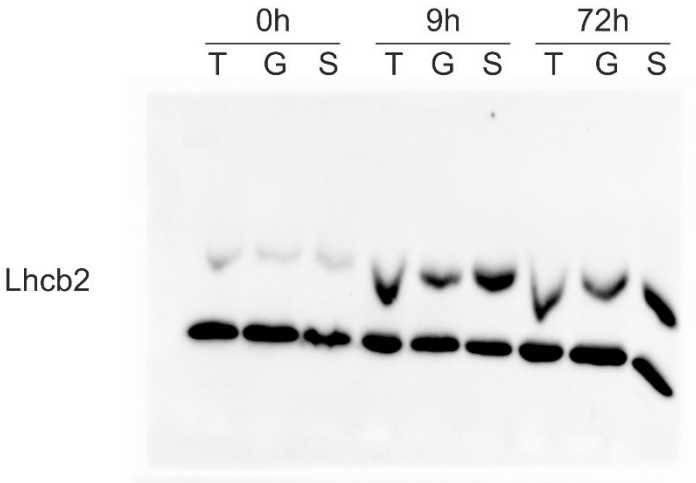

**Fig 7A**

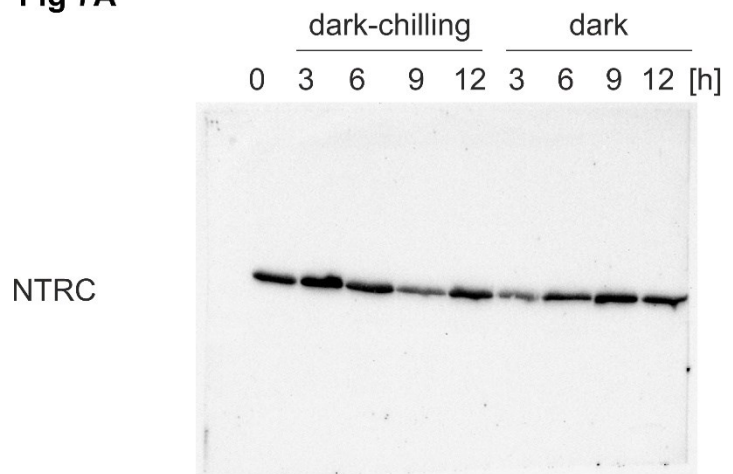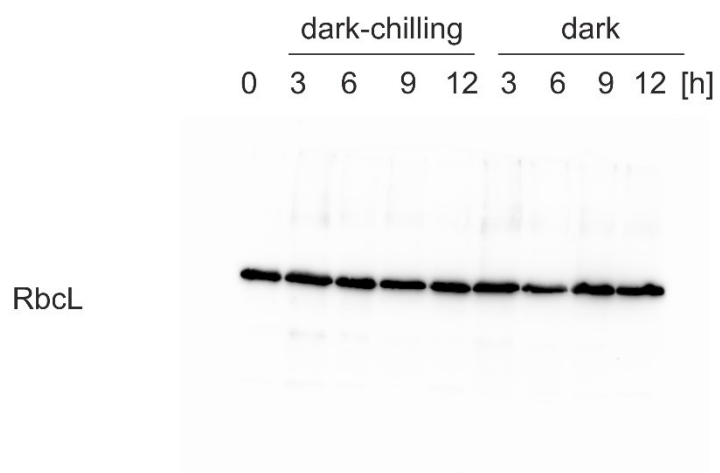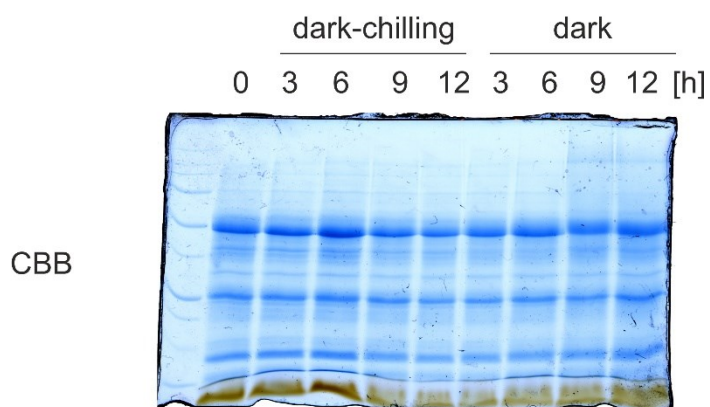

**Fig S3B**

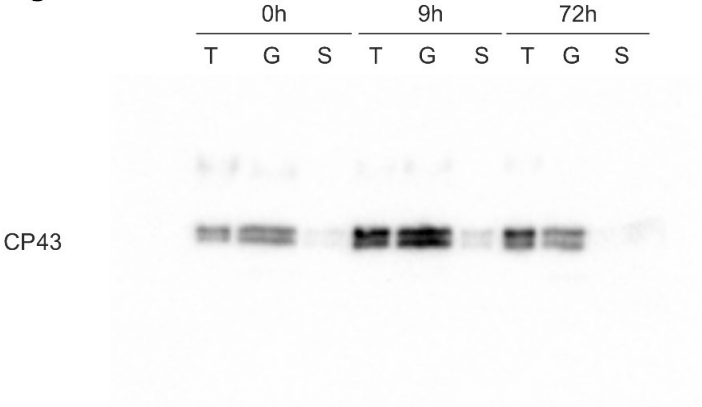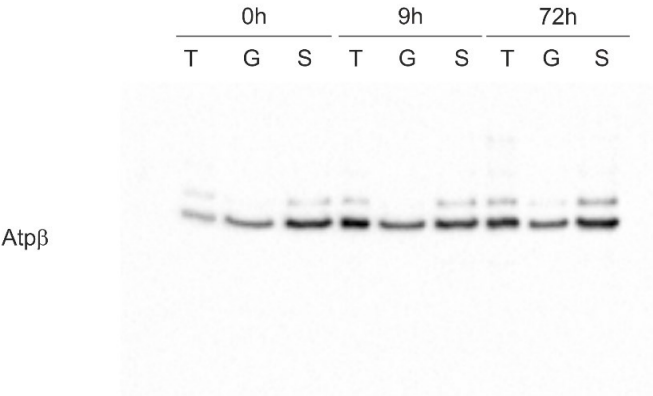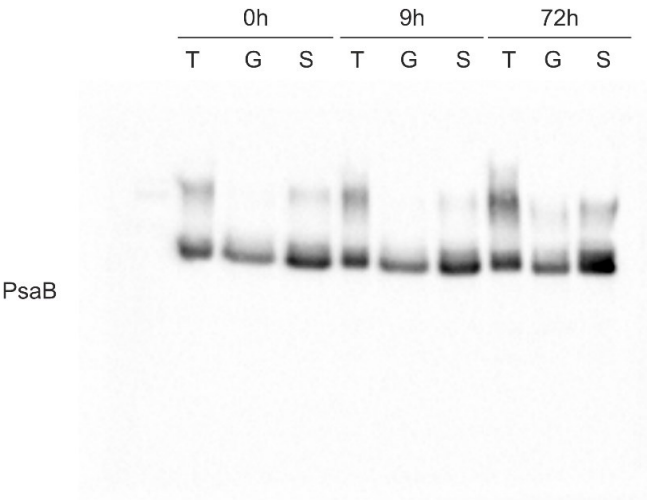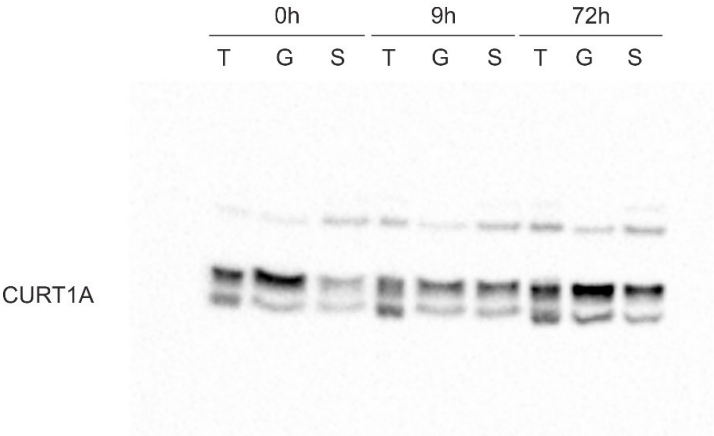

Supplement: Supplementary file 2 — Supplementary Material 2 [file 12870_2024_5169_MOESM2_ESM.pdf]
