# Supplementary material for: Light-independent pathway of STN7 kinase activation under low temperature stress in runner bean (Phaseolus coccineus L.)
Source: BMC Plant Biol. 2024 Jun 7;24:513. doi: 10.1186/s12870-024-05169-3 (PMC11157908; doi:10.1186/s12870-024-05169-3)
Supplement: Supplementary file 3 — Supplementary Material 3 [file 12870_2024_5169_MOESM3_ESM.pdf]

SUPPLEMENTARY MATERIAL

Light-independent pathway of STN7 kinase activation under low temperature stress in runner bean (*Phaseolus coccineus* L.)

Małgorzata Krysiak, Anna Węgrzyn, Łucja Kowalewska, Anna Kulik, Monika Ostaszewska-Bugajska, Jan Mazur, Maciej Garstka, Radosław Mazur*

* Author for correspondence: rmazur@uw.edu.pl

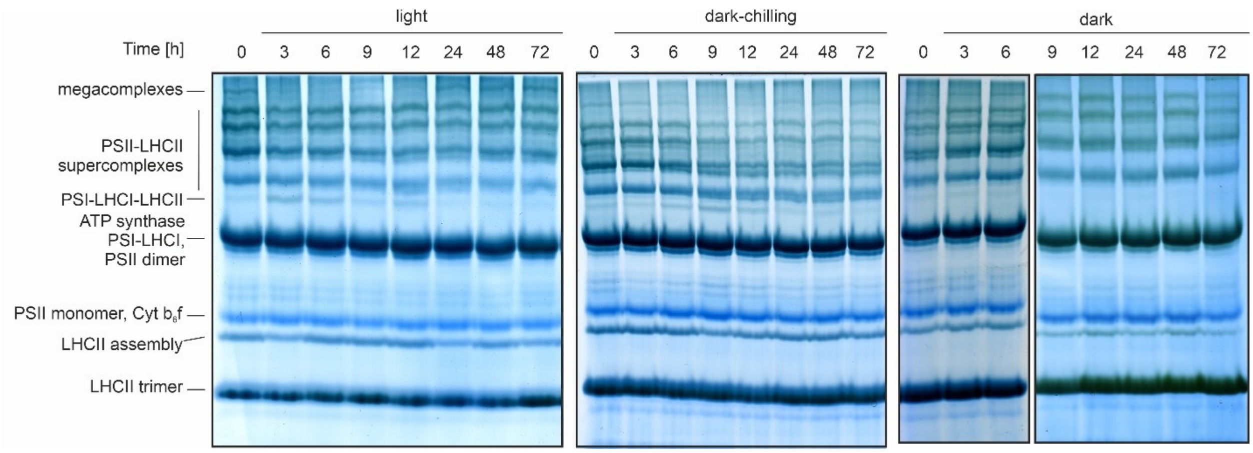

Fig. S1. Blue Native PAGE of runner bean's thylakoids exposed to light, dark-chilling and dark conditions. The presented gels are representative of 2 independent experiments.

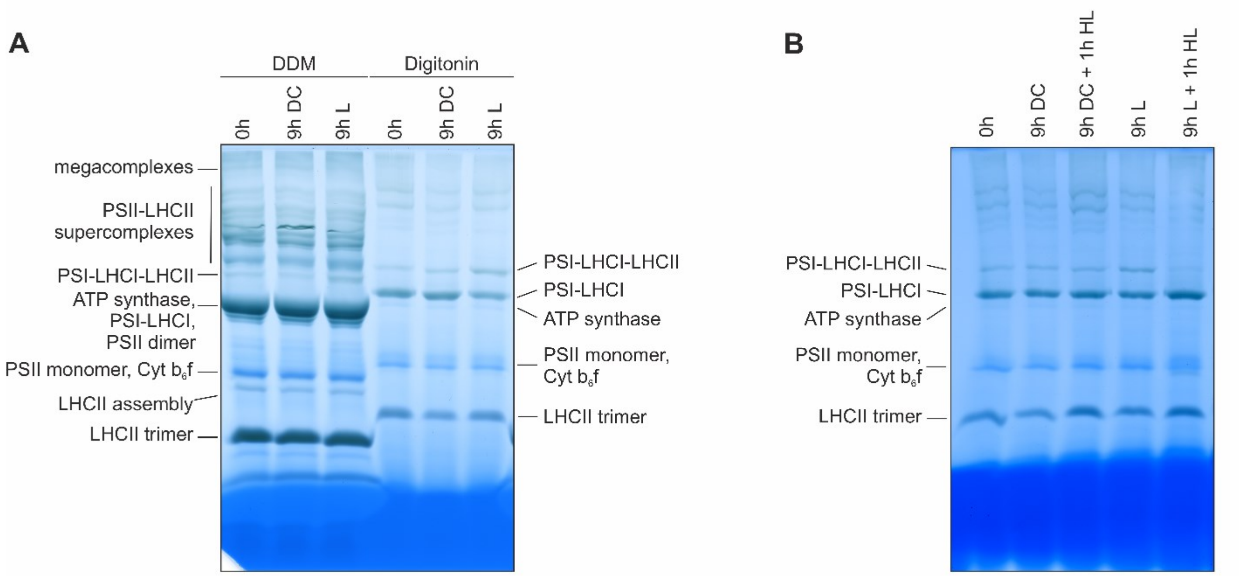

Fig. S2. Blue Native PAGE of runner bean's thylakoids exposed to 9 h of dark-chilling (DC) and growing light (L) conditions. (A) comparison between DDM and digitonin solubilization; the PSI-LHCI-LHCII complex is visible under both detergent treatments. (B) confirmation of PSI-LHCI-LHCII complex identity by exposure to high light (HL) of ~800 μmol photons m^{-2} s^{-1} during 1h; in both DC and L conditions, the PSI-LHCI-LHCII complex disappears after HL exposure only in L conditions. The presented gel is representative of two (A) or single (B) independent experiments.

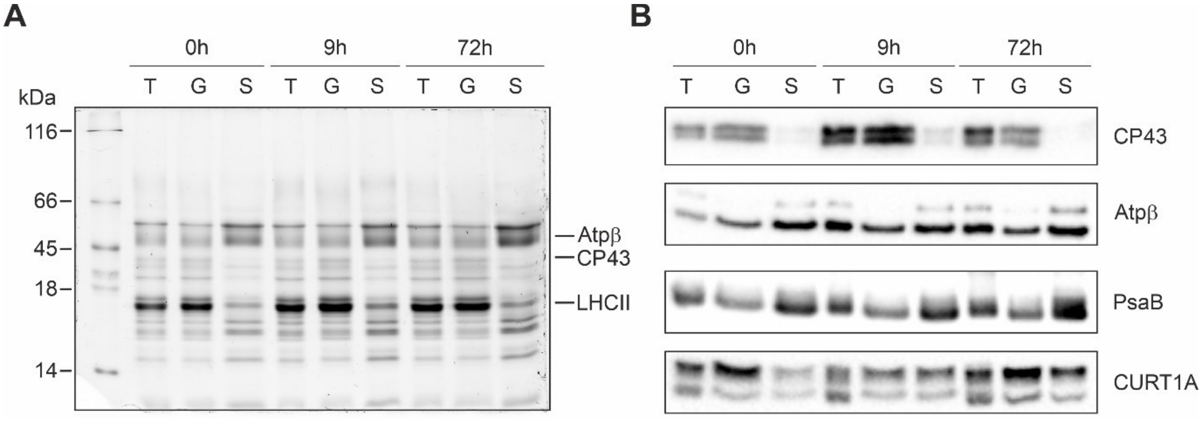

Fig. S3. Purity control of isolated thylakoid fractions. A – SDS PAGE of thylakoids (T) and thylakoid fractions: grana (G) and stroma lamellae (S) isolated from runner bean plants exposed to 0, 9, and 72 hours of dark-chilling. Gel was stained with SYPRO™ Ruby Protein Gel Stain. B – Immunoblots confirming the purity of obtained fractions performed for specified proteins. The presented gels and blots are representative of 3 independent experiments.

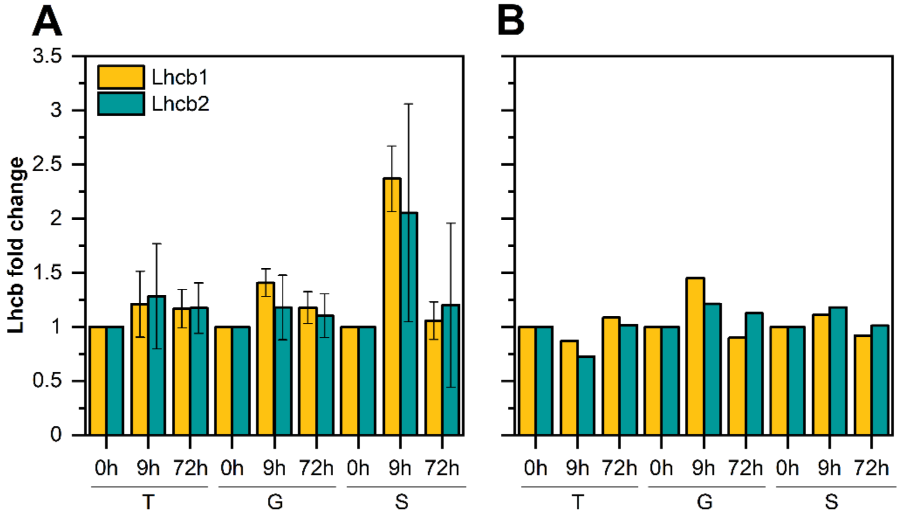

Fig. S4. Changes in Lhcb1 and Lhcb2 content in thylakoids (T) and thylakoid fractions: grana (G) and stroma (S) lamellae isolated from runner bean plants exposed to 0, 9, and 72 h of dark-chilling (A) or dark (B) conditions. Lhcb1 and Lhcb2 contents were calculated as a sum of upper and lower bands (see Fig. 4B), and the results were normalized to 0 h. The data are mean values ± SD from three (dark-chilling) or single (dark) independent experiments. Statistical analysis showed no significant differences between samples.

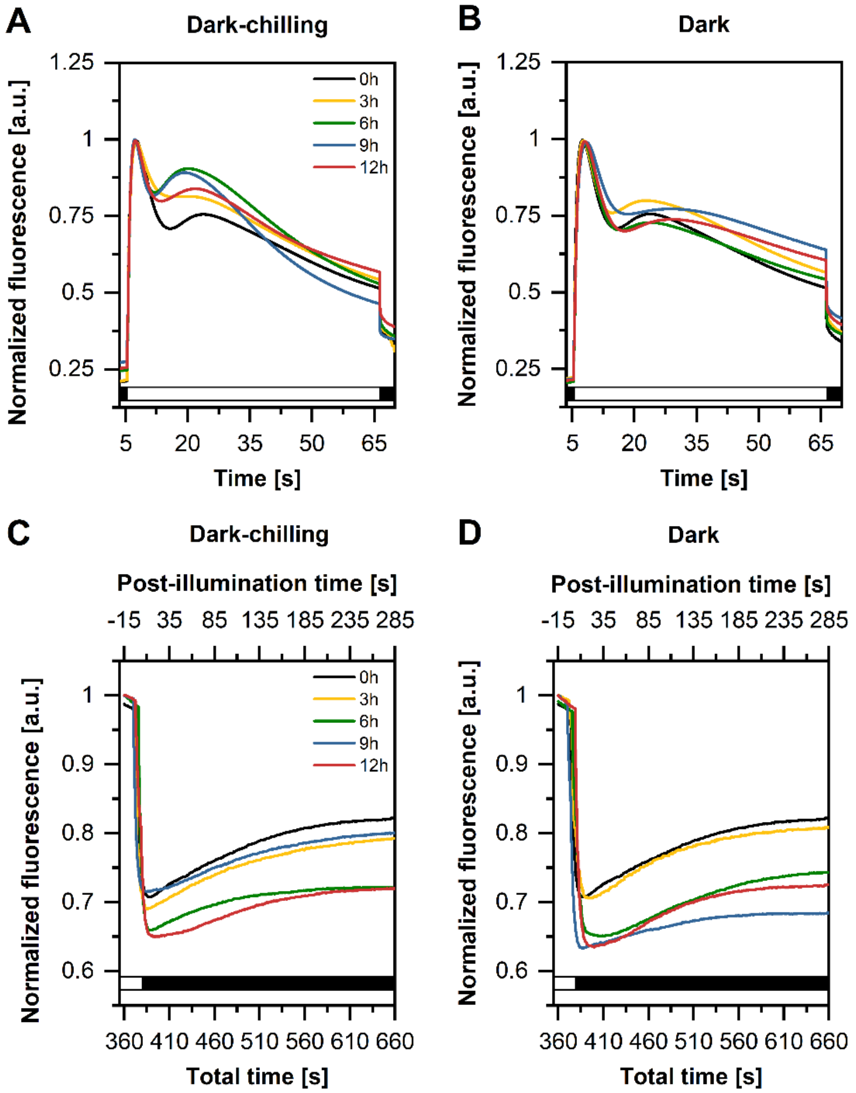

Fig. S5. Slow kinetics analysis of Chl *a* fluorescence *in vivo* in bean plants exposed to dark-chilling and dark conditions. (A-B) Chl *a* fluorescence changes during illumination with weak actinic light intensity in bean plants from dark-chilling (A) and dark (B) conditions. (C-D) Chl *a* fluorescence changes after actinic illumination in bean plants from dark-chilling (C) and dark (D) conditions. A fluorescence increase during the dark period indicates a non-photochemical reduction of the PQ pool. The exemplary fluorescence curve for the full measurement is presented in Fig 5F.

White and black bars on plots represent the illumination and dark phase of the measurement, respectively. The curves are mean values of at least 4 replicates (see Fig. 5 for details) from two independent experiments.

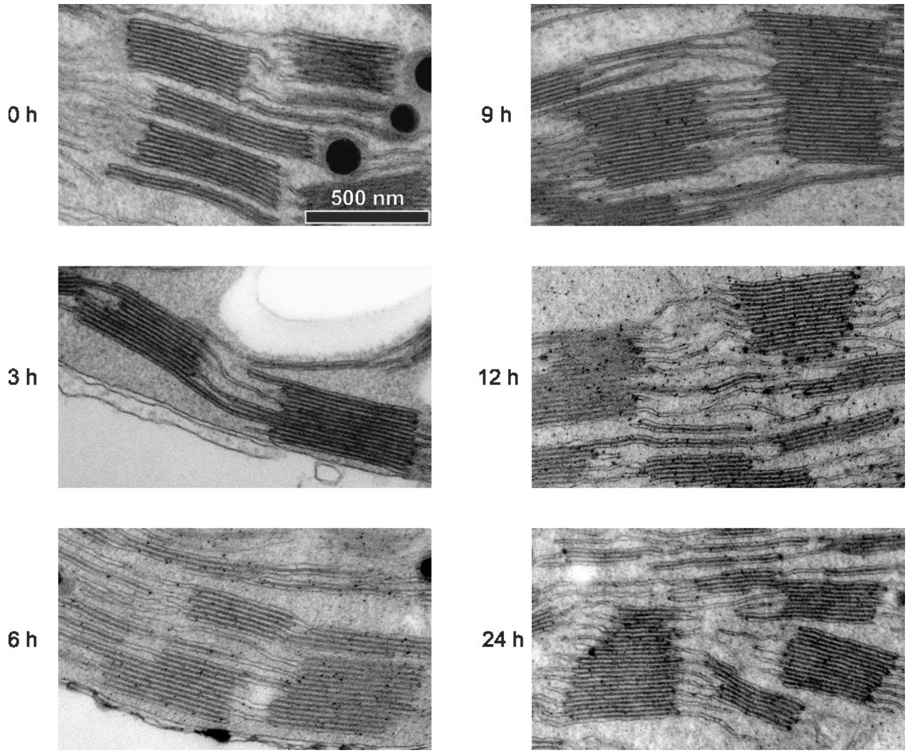

Fig. S6. TEM images showing grana structures of runner bean leaves under dark chilling conditions 0-24 h.

Table S1. List of used primary antibodies

Antibody	Agrisera Catalog Number
Lhcb1	AS01 004
Lhcb2	AS01 003
Lhcb1-P	AS13 2704
Lhcb2-P	AS13 2705
D1	AS10 704
CP43	AS11 1787
Lhca2	AS01 006
PsaA	AS06 172
PsaB	AS10 695
Atpβ	AS05 085
CURT1A	AS08 316
RbcL	AS03 037
NTRC	AS07 243

Table S2. List of primers for RT-qPCR

PvSkip16-F	CACCAGGATGCAAAAGTGG
PvSkip16-R	ATCCGCTTGTCCCTTGAAC
PvIDE-F	GCAACCAACCTTTCATCAGC
PvIDE-R	AGAAATGCCTCAACCCTTTG
PvNTRC-F	CTTGACCGCTCTGGCTATGT
PvNTRC-R	CTCCATTCATGGTCCTGCACA
